# Brd4 Regulates the Homeostasis of CD8^+^ T-Lymphocytes and Their Proliferation in Response to Antigen Stimulation

**DOI:** 10.3389/fimmu.2021.728082

**Published:** 2021-08-26

**Authors:** Zhilin Peng, Yiwen Zhang, Xiancai Ma, Mo Zhou, Shiyu Wu, Zheng Song, Yaochang Yuan, Yingshi Chen, Yuzhuang Li, Guanwen Wang, Feng Huang, Yidan Qiao, Baijing Xia, Weiwei Liu, Jun Liu, Xu Zhang, Xin He, Ting Pan, Hanshi Xu, Hui Zhang

**Affiliations:** ^1^Key Laboratory of Tropical Disease Control of Ministry of Education, Institute of Human Virology, Guangdong Engineering Research Center for Antimicrobial Agent and Immunotechnology, Zhongshan School of Medicine, Sun Yat-sen University, Guangzhou, China; ^2^Guangdong Provincial People’s Hospital, Guangdong Academy of Medical Sciences, Guangzhou, China; ^3^Bioland Laboratory (Guangzhou Regenerative Medicine and Health Guangdong Laboratory), Guangzhou, China; ^4^Center for Infection and Immunity Studies, School of Medicine, Sun Yat-sen University, Shenzhen, China; ^5^Department of Rheumatology, The First Affiliated Hospital, Sun Yat-sen University, Guangzhou, China

**Keywords:** CD8^+^ T cell homeostasis, CD8^+^ T cell proliferation, Brd4, glucose metabolism, antiviral immunity

## Abstract

CD8^+^ T cells are major components of adaptive immunity and confer robust protective cellular immunity, which requires adequate T-cell numbers, targeted migration, and efficient T-cell proliferation. Altered CD8^+^ T-cell homeostasis and impaired proliferation result in dysfunctional immune response to infection or tumorigenesis. However, intrinsic factors controlling CD8^+^ T-cell homeostasis and immunity remain largely elusive. Here, we demonstrate the prominent role of Brd4 on CD8^+^ T cell homeostasis and immune response. By upregulating Myc and GLUT1 expression, Brd4 facilitates glucose uptake and energy production in mitochondria, subsequently supporting naïve CD8^+^ T-cell survival. Besides, Brd4 promotes the trafficking of naïve CD8^+^ T cells partially through maintaining the expression of homing receptors (CD62L and LFA-1). Furthermore, Brd4 is required for CD8^+^ T cell response to antigen stimulation, as *Brd4* deficiency leads to a severe defect in clonal expansion and terminal differentiation by decreasing glycolysis. Importantly, as JQ1, a pan-BRD inhibitor, severely dampens CD8^+^ T-cell immune response, its usage as an anti-tumor agent or latency-reversing agent for human immunodeficiency virus type I (HIV-1) should be more cautious. Collectively, our study identifies a previously-unexpected role of Brd4 in the metabolic regulation of CD8^+^ T cell-mediated immune surveillance and also provides a potential immunomodulation target.

## Introduction

T-cell homeostasis maintenance is important for protective immunity and facilitates effective recognition of diverse antigens. Altered T-cell homeostasis frequently results in a dysfunctional immune response to infections and tumors, as well as the development of autoimmunity (1). Thus, the normal maintenance of CD8^+^ T-cell homeostasis is critical for the host to execute immune surveillance and prevent autoimmune diseases. Emerging evidence indicates that signals from TCR or interleukin-7 receptor (IL-7R) are critical for naïve T cell homeostasis (2–4). Besides, naïve T cells continually recirculate between peripheral circulation and secondary lymphoid organs to receive stimulation by homeostatic cues, which enhance T-cell survival and homeostasis proliferation (3). Thus, homing to secondary lymphoid organs is a prerequisite for access to IL-7 and self-peptide stimulation. Homing receptors, including CD62L, CCR7, and LFA-1, are responsible for naïve T cells transmigrating from circulation into lymph nodes (5). Transcription factors Foxo1 and Klf2 maintain peripheral naïve T-cell homeostasis by regulating migration receptor expression, including CD62L, CCR7 and S1PR1, to license naïve T cells for normal trafficking and survival (6–8). Moreover, energy production in mitochondria is also required for constant naïve T-cell migration (9).

Different metabolic pathways are employed to meet the bioenergetic and biosynthetic requirements for fulfilling the functions of distinct T-cell subsets. Naïve CD8^+^ T cells largely depend on oxidative phosphorylation, while effector CD8^+^ T cells preferentially use glycolysis (10, 11). Naïve CD8^+^ T cells mainly oxidize glucose in mitochondria to generate ATP for supporting cell migration and survival. Thus, decreased mitochondrial oxidative phosphorylation is tightly related to impaired survival and migration of naïve T cells. Deficiency of AIF (apoptosis inducing factor), a mitochondria-associated protein, causes a pronounced loss of peripheral naïve T cells through disrupting mitochondria respiration chain complex (12). Chemokine receptors CXCR4 and S1PR1 direct T-cell migration and maintain mitochondrial ATP production to support migration and survival (13, 14). Upon activation, CD8^+^ T cells increase glucose uptake and initiate metabolic reprogramming. Quick induction of glycolysis fulfils T cells’ functional demands, including quick proliferation, cell growth, survival, and cytokine secretion (10). Numerous reports suggest that CD8^+^ T cells cultured with limited glucose impairs proliferation, survival, and effector function (15–17). Transcription factor Myc is quickly elevated by TCR signal to induce glycolysis, which building blocks are generated to meet the demands of quick proliferation and effector differentiation (18).

Epigenetic reader Brd4 is involved in many biological processes, including gene expression, DNA damage response, and chromatin structure (19, 20). Increasing evidence suggests that *Brd4* deficiency affects the immune response, as evidenced by impaired B cell antibody class switching (21), defective macrophage development, and inflammatory response (22, 23), as well as impaired Th17 differentiation (24–26) and blocked ISP thymocyte development (27). In addition, targeting Brd4 has shown great potential for tumor eradication by downregulating Myc expression, particularly in hematopoietic malignancy (28, 29). Although the critical role of Myc in regulating glucose metabolism in tumor and T cells (18, 30) is well-studied and Brd4 inhibitors are broadly applied in HIV-1 latency reversal and cancer therapy (31–36), it is unclear whether Brd4 inhibition affects CD8^+^ T-cell responses. In this study, we aim to elucidate the role of Brd4 in CD8^+^ T-cell function. Therefore, we constructed mice with a specific deletion of Brd4 in T cells and studied its. We initially examined whether Brd4 gets involved in regulating CD8^+^ T-cell homeostasis in steady-state by analyzing CD8^+^ T cell counts and compartments. Then the CD8^+^ T cells response to antigen stimulation was investigated upon *Brd4* deletion during viral infection. Finally, small molecules targeting Brd4 were employed to explore the effect of Brd4 inhibition on CD8^+^ T cells function and differentiation. Throughout this analysis, we found Brd4 is required for the maintenance of naïve CD8^+^ T cell homeostasis and the efficient proliferation and differentiation of CD8^+^ T cells in response to antigen stimulation. By performing transcriptome analysis and metabolic prolife, we uncovered the central function of Brd4 in promoting naïve CD8^+^ T cells trafficking and glucose metabolism. The loss of Brd4 impairs the glucose uptake and homing of naïve CD8^+^ T cells. Impaired glucose uptake results in elevated apoptosis of naïve CD8^+^ T cells by decreasing oxidative phosphorylation, which synergizes with crippled homing to disrupt naïve CD8^+^ T cell homeostasis. In addition, the inefficiency of glucose uptake further limits CD8^+^ T cell proliferation due to poor glycolysis induction upon activation. Moreover, targeting Brd4 with inhibitors severely impairs CD8^+^ T cell function during viral infection. Mechanical study demonstrated that Brd4 directly promotes glucose transporter GLUT1 expression or the Brd4-Myc-GLUT1 axis indirectly controls glucose transporter GLUT1 expression. Together, these observations identified the key role of Brd4 in the regulation of CD8^+^ T-cell homeostasis and immunity and had implications for the inhibitor of Brd4 in therapeutic application.

## Material and Methods

### Animals and Cell Lines

C57BL/6 background floxed *Brd4^fl/fl^* mice were constructed through homologous recombination using a targeting vector containing loxP sites that flanked exon 3 of the *Brd4* locus. To achieve a specific deletion of *Brd4* in T cells, we crossed *Brd4^fl/fl^* mice with *CD4^cre^* mice. CD45.1, CD45.2, Rag1, *P14*×*Brd4^+/+^*, and *P14*×*Brd4^-/-^* transgenic mice were used in adoptive transfer experiments. All mice used in experiments were 6 to 8 weeks old and housed under SPF conditions in the animal center of Sun Yet-Sun University. Littermate mice were used as controls. All mouse experimental procedures were approved by the Institutional Animal Care and Use Committee of Sun Yet-Sen University. BHK21 and Vero-E6 cells were purchased from ATCC and cultured in DMEM; they were verified to be mycoplasma-free.

### Virus Production, Titration, Infection, and Adoptive Transfer

The Armstrong strain of LCMV was kindly provided by Prof. Lilin Ye (Third Military Medical University) and was grown in BHK21 cells and harvested at 48 h post-infection. The virus titer was determined by calculating the plaque forming units. Cognate distinct CD45.1 and CD45.2 mice were used in adoptive transfer experiments. Naïve P14^+^CD8^+^ T cells from *Brd4^+/+^*and *Brd4^-/-^* mice were isolated by magnetic beads (Stemcell). Then, 5×10^3^ CD45.2^+^ CD45.1^+^ *Brd4^+/+^* P14^+^ CD8^+^ T cells and 5×10^3^ CD45.2^+^ CD45.1^-^ *Brd4^-/-^* P14^+^ CD8^+^ T donor cells were mixed at a 1:1 ratio and transferred into CD45.2^-^CD45.1^+^ recipient mice. One day later, recipient mice were infected through intraperitoneal injections with 2 × 10^5^ PFU LCMV-Arm.

### Antibody and Flow Cytometry

The following fluorochrome-conjugated antibodies were used for analysis: anti-CD3 (clone:145-2C11), anti-CD8α (clone:53-6.7), anti-CD4 (clone:GK1.5), anti-CD44 (clone:IM7), anti-CD62L (clone:MEL-14), anti-KLRG1 (clone:2F1), anti-CD127 (clone:A7R43), anti-CD45.1 (clone:A20), anti-CD45.2 (clone:104), anti-CD71 (clone:R17217), anti-CD27 (clone:LG.3A10), anti-IFNγ (clone:XMG1.2), anti-IL2 (clone:JES6-5H4), anti-Myc (clone:SH1-26E7.1.3), anti-BCL-2 (clone:10C4), and LCMV-gp33 Tetramer (MBL). MHC-I tetramer staining was completed at 4°C for 45-60 minutes according to the manufacturer’s instructions. Dead cells were excluded before analysis with Live/Dead dye staining (ThermoFisher). For intracellular cytokine staining, prepared single-cell suspensions were stimulated for 4-6 hours at 37°C with gp33 peptide in the presence of GolgiStop™ and GlolgiPlug™ (BD Bioscience). Before intracellular staining, surface staining was performed in 4°C for 30 minutes. Then, cells were fixed and permeabilized by using the Intracellular Fixation & Permeabilization Buffer Set (Invitrogen) and intracellular cytokine staining was performed with the indicated antibodies at room temperature for 30 minutes. For intracellular transcription factor staining, Foxp3/Transcription Factor Staining Buffer Set (Invitrogen) was used according to the manufacturer’s instructions. All flow cytometry was performed with a BD LSRFortessa and analyzed with FlowJo software.

### *In Vitro* Stimulation, Proliferation, and Cell Death Measurement of CD8^+^ T Cells

Naïve CD8^+^ T cells from *Brd4^+/+^*and *Brd4^-/-^* mice were activated *in vitro* with plate-bound anti-CD3 (5 µg/mL) and anti-CD28 (2 µg/mL) antibodies, as well as IL-2 (100 U/mL) for 48 hours. To determine cell proliferation, *Brd4^+/+^*and *Brd4^-/-^* naïve CD8^+^ T cells were labeled with cell proliferation dye CTV (5 μM) at 37°C for 25 minutes and activated for 3 days. For the measurement of cell death, intracellular active caspase3/7 (ThermoFisher) and Annexin V/PI (ThermoFisher) surface staining was performed and evaluated through flow cytometry.

### *In Vivo* Administration of BET Inhibitor JQ1

For the LCMV acute infection mouse model, *Brd4* inhibitor JQ1 was intraperitoneally injected into 8-week-old wild-type C57BL/6 mice at 50 mg/kg before infection. One day later, the mice were challenged with 2 × 10^5^ PFU LCMV-arm strain and injected with same dose of JQ1 inhibitor again. After infection, the mice received daily JQ1 administration until day 8 post-infection. At day 9 post-infection, all mice were sacrificed for further analysis.

### RNA-Seq Analysis and Quantitative RT-PCR

Naïve CD8^+^ T cells were isolated from the spleen and lymph nodes with FACS sorting. Purified naïve CD8^+^ T cells were stimulated with anti-CD3/CD28 plus with rhIL-2 for 18h. Naïve and activated CD8^+^ T cells were lysed by TRIzol Reagent (ThermoFisher) and RNA were extracted as per the manufacturer’s instruction. Each RNA-seq sample includes three biologic replicates. The quality and quantity of RNA was assessed with Nanodrop 2000 (ThermoFisher) and BioAnalyzer 2100 (Aglient). RNA-seq library was generated and sequenced by Shanghai Biotechnology Corporation. RNA-Seq reads were trimmed, filtered, and quality-controlled by FastQC (Babraham Institute) tool. The reads were aligned to mouse reference genome NCBI build 38 (GRCm38) by Hisat2, followed by calculating the reads per kilobase per million mapped reads (RPKM). Differentially-expressed genes (FDR < 0.05, FC > 2) were determined by EdgeR (v3.28.0). GSEA was performed with ClusterProfiler (v3.14.0). Heat maps were plotted with the R package ggplot2. Bigwig files were generated with STAR (v2.4.2).

Total RNA of target cells was extracted with TRIzol reagent (ThermoFisher) and subjected to cDNA synthesis with HiScript II 1st Strand cDNA Synthesis Kit (Vazyme). Gene expression was evaluated by real-time qPCR with SYBR Master Mix (Vazyme) in a CFX96 Real-time PCR Detection System (Bio-Rad). Mouse β-actin mRNA expression was used as a loading control.

### Cleavage Under Targets and Tagmentation (CUT&Tag)

Fifty-thousand isolated CD8^+^ T cells were used to generate CUT&Tag library as a reference (37). First, CD8^+^ T cell was incubated with primary antibody against *Brd4* (1:50; Active motif AB_2615059) and then with guinea pig anti-rabbit secondary antibody (1:100; Antibodies Online ABIN101961). This was followed by adding the prepared pA-Tn5 complex. Next, DNA was fragmented by Mg2+ activator and the resulting DNA segments were extracted to amplify with PCR. Finally, PCR products were cleaned up and sequenced. Paired-end 150-bp sequencing was performed on an Illumina HiSeq 2500. Each DNA library for CUT&Tag was sequenced with HiSeq 2500 system (Illumina, USA) under the paired-end 150 bp mode. For data processing, raw sequencing data were trimmed and filtered by using Trim Galore. The paired-end reads with high quality (Q30) were aligned to the mouse reference genome mm10 using the Burrows Wheeler Aligner with default parameters and then sorted using the SAMtools software. The “MarkDuplicates” function embedded in Picard was used to mark and discard PCR duplicates. For comparison between different samples, mapped reads were down-sampled to equalize reads across samples by using Samtools. MACS2 was used to quantify the Brd4 CUT&Tag signal throughout the genome and within gene bodies.

### Chromatin Immunoprecipitation Quantitative PCR Analysis

Chromatin immunoprecipitation (ChIP) assays were performed according to the manufacturer’s instructions using the ChIP kit (Cell Signaling Technology). Briefly, 4,000,000 purified CD8^+^ T cells were fixed with 1% formaldehyde for 10 minutes at room temperature, and the fixation was quenched with glycine at a final concentration of 125 mM. Chromatin was digested with micrococcal nuclease into 150–900-bp DNA/protein fragments. Then, 10 μg of digested, cross-linked chromatin was used for each ChIP experiment. Two micrograms of Abs (Active motif) against BRD4 were used for each assay. ChIP–quantitative PCR (qPCR) primer for *Slc2a1* and *Myc* in this study were as follows: *Slc2a1* forward, 5′- GAACTTAATGCCACTTTACACATA -3′ and *Slc2a1* reverse, 5′- TAGGACTCTGGCTTCCTCACTAGGC-3′. *Myc* forward, 5′-TAAAAGGGGAAAGCTTGGGTTTGTC-3′ and *Myc* reverse, 5′-GAGAATATGCCATGAATTGGGGGGTT-3′.

### Western Blotting

CD8^+^ T cells were lysed with RIPA buffer to prepare cell lysate samples. Protein extracts were resolved by 4–12% SDS-PAGE (Invitrogen), then were transferred to a polyvinylidene fluoride membrane (Millipore) and analyzed by immunoblotting with anti-Brd4 (EPR5150, Abcam) and anti-β-actin (20536-1-AP, Proteintech) antibodies.

### Glucose Uptake

Glucose uptake was evaluated directly by using fluorescent glucose analog 2-NBDG(Abmole). One million naïve or stimulated CD8^+^ T cells were incubated with 2-NBDG at a final concentration of 100 μM in glucose-free culture media for 30 minutes at 37°C. After that, intracellular 2-NBDG was assessed with flow cytometry.

### Metabolic Profiling

CD8^+^ T-cell oxygen consumption rate (OCR) and extracellular acidification rate (ECAR) were measured by using a Seahorse XFe96 Extracellular Flux Analyzer. Briefly, naïve or stimulated CD8^+^ T cells were plated in poly-D-lysine coated XF96 plates *via* transient centrifuge in XF media (Agilent) to firmly adhere to culture plate. OCR was measured under basal conditions and after sequential treatment with following compounds: ATP synthase Oligomycin (1 μM), protonophore carbonyl cyanide-4-phenylhydrazone (FCCP) (1.5 μM) to uncouple mitochondria, and mitochondrial complex I inhibitor rotenone (100 nM) and mitochondrial complex III inhibitor antimycin A (2 μM) to suppress mitochondrial respiration. Indices of mitochondrial respiration were calculated from OCR profile: basal OXPHOS (Basal OX), calculated as the difference of basal OCR and OCR induced by rotenone + antimycin, and maximum respiratory capacity (MRC), calculated as the difference of OCR induced by FCCP and OCR induced by rotenone + antimycin. ECAR was evaluated under basal conditions and with sequential addition of 10 mM glucose, 1 μM oligomycin, and 100 mM of 2-DG. Indices of glycolysis were calculated from the ECAR profile: glycolysis (glyc), calculated as the difference of ECAR induced by glucose and ECAR induced by 2-DG, and maximal glycolytic capacity (MGC), calculated as the difference in ECAR induced by oligomycin and that induced by 2-DG.

### *In Vitro* Chemokine-Driven Migration

Migration assays were performed in 24-well Trans-well plates containing polycarbonate filters (5-µm pore size, Corning), according to a previous work with minor modifications (38). Briefly, isolated naïve CD8^+^ T cells were added to the top well, while the bottom receiver well was filled with RPMI-1640 medium supplementing with 500 ng/ml CCL21 to drive naïve CD8^+^ T cells migration. After chemotaxis for 4 h, the filter plate was removed, EDTA was added to each well (40 mM final concentration) and the cells were transferred to 96-well V-bottom plates, spun, resuspended in PBS/- 1% FBS, and analyzed on a flow cytometer. The percentage of migration was calculated by dividing the number of cells that migrated through the filter by the total number of cells that added to each well.

### shRNA-Mediated Knockdown by Retroviral Transduction

The shRNAs targeting mouse LFA-1 were subcloned into retroviral vectors containing GFP open reading frame (ORF) (pMKO.1-GFP). Retrovirus particle was packaged by co-transfecting shRNA-expressing retroviral vector and pCL^eco^ into Plate-E cells. After 48 h, retroviral particle was harvested and stored at 80°C. Naïve CD8^+^ T cells were purified from *Brd4^+/+^* mice and activated with 5 µg/ml plate-bound anti-CD3 and 2 µg/ml anti-CD28 plus 100 U/ml rhIL-2 for 24 h. After activation, CD8^+^ T cells were infected by retrovirus particle supplemented with 100 U/ml rhIL-2 and 8 µg/ml polybrene, followed by centrifugation for 120 min at 800 g at 35°C. After incubation for another 6 h at 37°C, supernatant of transduced CD8^+^ T cells were replaced with fresh culture medium and cultured for another 2-3 days for cell proliferation analysis.

### *In Vivo* Killing Assay

*In vivo* killing assay was carried out as previously described (39). Briefly, splenocytes from naïve C57BL/6J (CD45.2) mice were labelled with CFSE (Lifetechnologies) at either 100 nM (CFSE^lo^) or 1 μM (CFSE^hi^). The labelled cells (CFSE^lo^; specific target cells) were then pulsed with 2 μg of LCMV-GP33-41 peptides for 1 h at 37°C and then rinsed three times in RPMI 1640 with 10% FBS. The peptide pulsed target cells (CFSE^lo^; specific target cells) and non-pulsed cells (CFSE^hi^; non-specific target cells) were mixed at a 1:1 ratio. Then sorted *Brd4^+/+^* or *Brd4^-/-^* P14^+^CD8^+^ T cells from same infection recipient mice were added into the mixed target cells at a 1:2 effector: target ratio (E: T), followed by transferring into naïve C57BL/6J (CD45.1) mice. Mice were euthanized for analysis of target cell percentage 5 h later.

### Statistical Analysis

Statistical significance was calculated by using GraphPad Prism 8. Before testing the statistical difference, the normality and equal variance were firstly checked to ensure reliability of data analysis. *P* value between two independent groups was analyzed by unpaired Student’s *t-*test, while paired Student’s *t*-test was used when the samples being compared originated from the same mouse. In addition, *P* value among multiple groups was calculated by parametric one-way ANOVA. All results are represented as mean ± s.e.m. In figures, asterisks denote statistical significance (NS, not significant; *p < 0.05; **p < 0.01; ***p < 0.001; ****p < 0.0001).

### Data Availability

All raw sequencing data generated in this study are available within the paper. The RNA-seq and CUT&Tag-seq data have been deposited in the Gene Expression Omnibus (GEO) under accession number GSE179492. Additional information and materials will be made available upon request.

## Results

### Loss of *Brd4* Impairs Peripheral T-Cell Homeostasis

To determine the function of *Brd4* in CD8^+^ T cells, we analyzed the dynamic expression of *Brd4* in CD8^+^ T-cell differentiation. Data from public databases indicate that the expression of *Brd4* is differentially upregulated in the effector differentiation of CD8^+^ T-cell during acute or chronic infection (**Supplemental Figure 1A**). Moreover, the expression of *Brd4* is quickly induced by *in vitro* TCR signal stimulation in CD8^+^ T cells (**Supplemental Figure 1B**). These preliminary data suggest that Brd4 is highly likely to regulate the function of CD8^+^ T cells. Considering the prominent role of Brd4 in HIV-1 latency and tumor therapy and the essential role of CD8^+^ T cells in antiviral/antitumor immunity, it is urgent to clearly evaluate the effect of Brd4 inhibition on CD8^+^ T cell function and differentiation *in vivo*.

**Figure 1:**
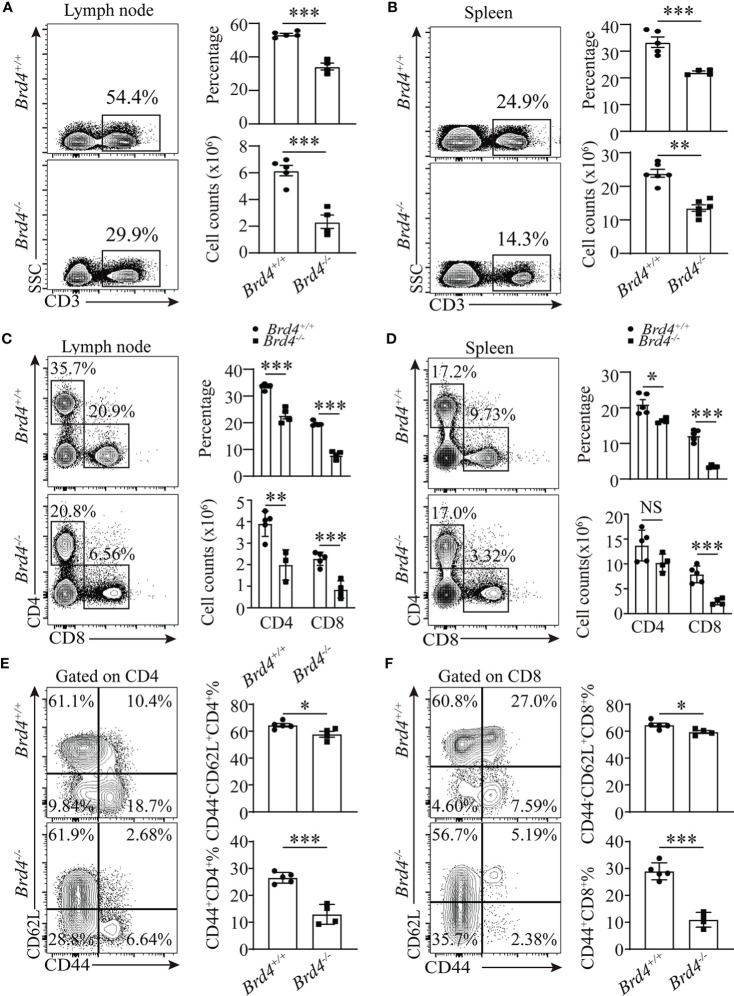
Brd4 is required for peripheral T cells maintenance. **(A, B)** Flow cytometry showing the expression of CD3 (left) and quantification of total CD3^+^ T cells (right) in lymph node **(A)** and spleen **(B)**. **(C, D)** CD4 and CD8 expression determined by flow cytometry in lymph node **(C)** and spleen **(D)** (left), the absolute number and proportion of CD4^+^ T cells and CD8^+^ T cells in spleen and lymph node (right). **(E, F) **Naïve (CD44^-^CD62L^+^) and memory/activated (CD44^+^) T cells compartment analysis within CD4^+^ T **(E)** and CD8^+^ T cells **(F)** from freshly prepared splenocytes. Two-tailed unpaired t-test was used to analyze two independent groups. Results were indicated as mean ± sem (error bars). *p < 0.05; **p < 0.01; ***p < 0.001. NS, not significant. Each group includes at least 3 mice and each experiment repeats more than 3 times.

To further investigate the role of *Brd4* in CD8^+^ T-cell function, we generated *lox*P-flanked *Brd4* allele mice (*Brd4^fl/fl^*) and then crossed this strain with *CD4^cre^* mice to obtain specific deletion of *Brd4* in T cell (**Supplemental Figures 1C, D**), hereafter referred to as knockout (*Brd4^-/-^*) mice, and the littermate wild-type mice referred to as (*Brd4^+/+^*) mice. Immunoblotting indicated that Brd4 was completely deleted in CD8^+^ T cells (**Supplemental Figure 1E**). Thymocyte development was firstly examined in *Brd4^-/-^* mice. Comparable thymocyte frequency and absolute number were observed in *Brd4^-/-^* and *Brd4^+/+^* mice (**Supplemental Figures 1F–I**), suggesting that Brd4 is not required for the development of late thymocytes. Normal thymocytes development observed in this study was consistent with previous work which showed that *Lck^cre^* rather *CD4^cre^* -mediated deletion of *Brd4* blocks thymus development (27). Next, we investigated whether *Brd4* deficiency affects peripheral T-cell homeostasis by enumerating T-cell frequency and absolute number in peripheral lymphoid tissues. A 50% reduction of absolute number and 40~50% reduction of relative frequency of CD3^+^ T-cell was detected both in the spleen and lymph nodes in *Brd4^-/-^* mice relative to *Brd4* ^+/+^ mice (**Figures 1A, B**). With this insight into CD3^+^ T-cell subsets, we found that *Brd4* deficiency caused approximately 2-fold reduction of CD8^+^ T cells in both spleen and lymph nodes, while a notable reduction of CD4^+^ T cells only in lymph nodes (**Figures 1C, D**). In addition, a consistent reduction of CD8^+^ T cells in peripheral blood was observed, even though the reduction was much less than that in lymphoid organs (**Supplemental Figure 1J**). We also noted a slight increase of CD4^+^ T cells in peripheral blood (**Supplemental Figure 1J**). There was no specific accumulation of T cells in non-lymphoid organ (liver) in *Brd4^-/-^* mice compared to *Brd4^+/+^* mice (**Supplemental Figure 1K**). These observations indicate that the peripheral lymphopenia in *Brd4^-/-^* mice barely results from defective development of thymocytes and increased accumulation of T cells in non-lymphoid tissues.

The overall reduction in T-cell number frequently results in changes in the peripheral T-cell compartments. We next analyzed T-cell populations in *Brd4^-/-^* mice in more detail by evaluating the expression of CD44 and CD62L. Compared to *Brd4^+/+^* mice, there was an approximately 60% reduction of CD44^+^CD62L^-^ (effector/memory) population in *Brd4^-/-^* mice, while a relative normal CD44^-^CD62L^+^(naïve) population among conventional CD4^+^ T and CD8^+^ T cells (**Figures 1E, F**). Taken together, these findings suggest that Brd4 is indispensable in the maintenance of peripheral T-cell homeostasis. Because CD8^+^ T cells are more sensitive to *Brd4* deletion, determined by the ratio of CD8 to CD4 (**Supplemental Figure 2A**), we focused on CD8^+^ T cells in the subsequent study.

### *Brd4* Deficiency Impairs the Survival and Homeostatic Proliferation of Naïve CD8^+^ T Cells

**Figure 2:**
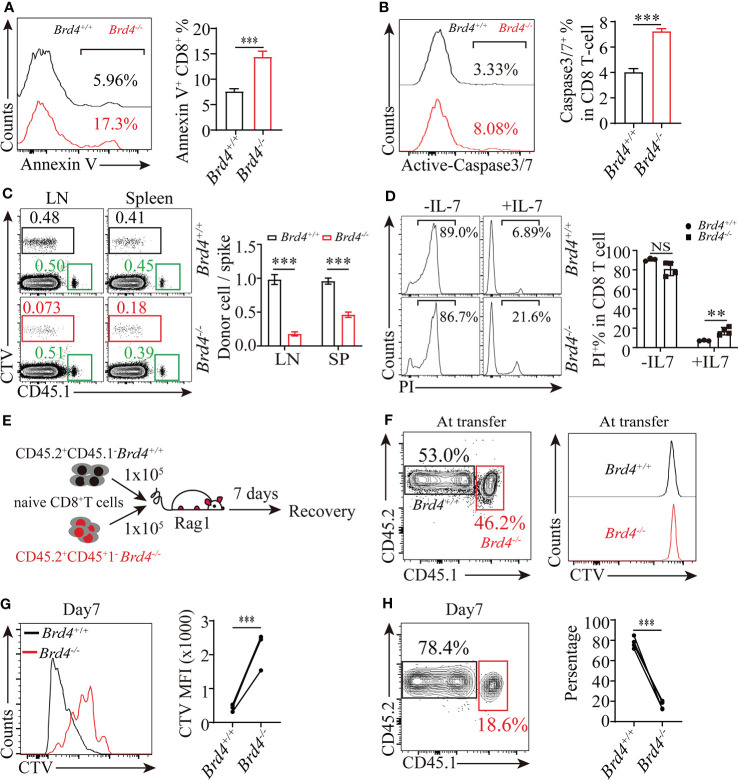
Brd4 is essential for naïve CD8^+^ T cells survival and homeostasis proliferation. **(A)** Flow cytometry showing the frequency (left) and quantification (right) of Annexin-V^+^ CD8^+^ T cells. **(B)** Flow cytometry showing the frequency (left) and quantification of active-caspase3/7^+^CD8^+^ T cells (right). **(C)** Purified *Brd4^+/+^* and *Brd4* ^-/-^ naïve CD45.2^+^CD8^+^ T cells were labeled with CellTrace Violet (CTV) and respectively transferred with naïve CD45.1^+^CD8^+^ T cells (spike) into CD45.2^+^ recipient mice to monitor the survival capability of naïve CD8^+^ T cells for 7 days *in vivo*. Flow analysis of donor naïve CD8^+^ T cells in spleen and lymph node at day 7 post-transfer (left) and the relative ratio of recovered donor cells to spike (right). **(D)** Sorted naïve CD8^+^ T cells from *Brd4^-/-^* and *Brd4^+/+^* mice were cultured with or without IL-7 for 3 days to test IL-7 triggered survival effect. **(E)** Experimental setup. Congenically marked *Brd4^+/+^* and *Brd4* ^-/-^ naïve CD8^+^ T cells were labeled with CTV, mixed at a 1:1 ratio, and then transferred into Rag1 mice to monitor cell proliferation at day 7 post-transfer. **(F)** Relative frequency of donor cells at transfer. **(G)** Representative flow analysis of donor cells recovered at day 7 post-transfer. **(H)** The quantification of donor CD8^+^ T cells at day 7 post transfer. Two-tailed unpaired *t*-test was used to analyze two independent groups, while paired Student’s *t*-test was used when sample being compared from same mouse. Results were indicated as mean ± sem (error bars). **p < 0.01; ***p < 0.001. NS, not significant. Each group includes at least 3 mice and each experiment repeats more than 3 times.

The pool and composition of peripheral T cells are determined by cell survival and basal steady-state homeostatic proliferation. To examine the cell survival of peripheral T cells, surface Annexin-V and intracellular active-caspase3/7 staining was used to evaluate apoptosis by flow cytometry. We observed approximately a 1-fold increase of surface Annexin-V and intracellular active-caspase3/7 in naïve CD8^+^ T cells from *Brd4^-/-^* mice, indicating that *Brd4* deletion increased cell apoptosis (**Figures 2A, B**). In addition, the survival capability of naïve CD8^+^ T cells was also determined *in vivo*. Equal numbers of *Brd4* ^-/-^ or *Brd4* ^+/+^ naïve CD8^+^ T cells labeled with CellTrace™ Violet (CTV) were mixed with equal numbers of naïve CD45.1^+^CD8^+^ T cells, which served as internal control. These mixtures were then transferred into CD45.2^+^ mice to monitor the survival of naïve CD8^+^ T cells (**Figure 2C**). In contrast to *Brd4^+/+^* naïve CD8^+^ T cells, which were stably maintained in recipient mice, *Brd4* ^-/-^ naïve CD8^+^ T cells failed to be efficiently maintained and rapidly declined to 20% and 50% in lymph nodes and spleen after 7 days post-transfer, respectively (**Figure 2C**). As IL-7 signal is crucial in the survival of naïve T cells, we next investigated whether the increased apoptosis results from defective IL-7 signal transduction. To this end, purified naïve CD8^+^ T cells were cultured in the presence or absence of IL-7 for 3 days. *Brd4^-/-^* CD8^+^ T cells were less responsive to the IL-7 stimulated survival signal than *Brd4^+/+^* CD8^+^ T cells (**Figure 2D**). However, comparable IL-7Rα (CD127) and BCL-2 expressions were detected after *Brd4* deletion, indicating that the impaired response to IL-7 was not caused by decreased expression of its receptor (**Supplemental Figures 2B, C**). Collectively, these results indicate that *Brd4* is required for naïve CD8^+^ T-cell survival.

We next determined homeostatic proliferation of CD8^+^ T cells after *Brd4* deletion. Because of severe lymphopenic microenvironment in *Brd4^-/-^* mice, which trigger abnormal homeostatic proliferation, these mice could not be used to examine Brd4 function in CD8^+^ T-cell homeostatic proliferation. To rule out the influence of different microenvironments in *Brd4^+/+^* and *Brd4^-/-^* mice, we isolated naïve CD8^+^ T cells from congenically distinct *Brd4^+/+^* and *Brd4^-/-^* mice, labeled them with CTV, and mixed them at a 1:1 ratio. Then, mixed naïve CD8^+^ T cells were adoptively transferred into Rag1 mice to evaluate homeostatic proliferation at day 7 post-transfer (**Figures 2E, F**). We observed that *Brd4^-/-^* CD8^+^ T cells underwent limited cell divisions, while *Brd4^+/+^* CD8^+^ T cells underwent 4~5 rounds division to an undetectable level in recipient Rag1 mice (**Figure 2G**). Meanwhile, *Brd4^+/+^* CD8^+^ T cells outcompeted *Brd4^-/-^* CD8^+^ T cells in relative frequency due to limited *Brd4^-/-^* CD8^+^ T-cell proliferation (**Figure 2H**). These results indicate that Brd4 is required for maintaining CD8^+^ T-cell homeostatic proliferation.

### Brd4 Supports Optimal Antigen-Specific CD8^+^ T-Cell Clonal Expansion During Infection

**Figure 3:**
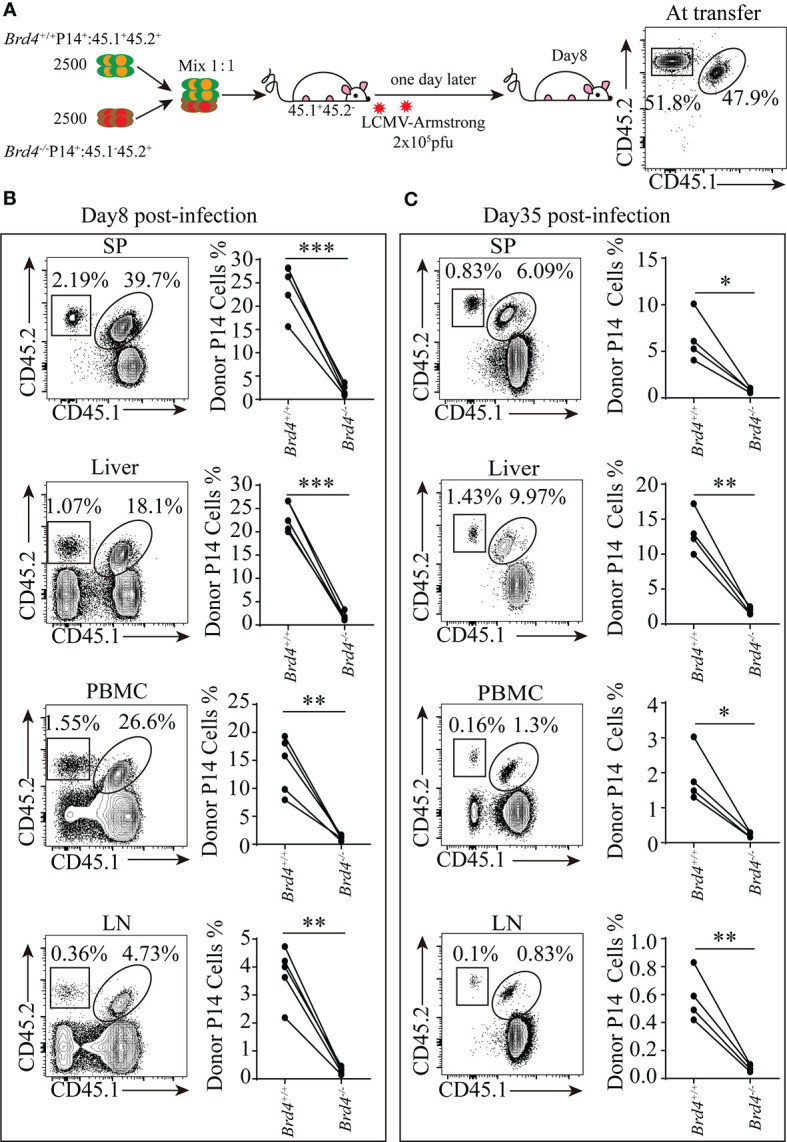
Brd4 promotes the clonal expansion of CD8*^+^* T cells during viral infection. **(A)** Experiment setup. **(B)** *Brd4^+/+^* and *Brd4^-/-^* effector P14^+^ CD8^+^ T cells recovered at day 8 in indicated tissues (left) and the quantification of effector P14^+^CD8^+^ T cells post-infection (right). **(C)** *Brd4^+/+^* and *Brd4^-/-^* memory P14^+^ CD8^+^ T cells recovered at day 35 in indicated tissues (left) and the quantification of memory P14^+^ CD8^+^ T cells post-infection (right). Paired Student’s *t*-test was used to analyze data. Results were indicated as mean ± sem (error bars). *p < 0.05; **p < 0.01; ***p < 0.001. Each group includes at least 4 mice and each experiment repeats more than 3 times.

*Brd4* deficiency not only results in a marked reduction of peripheral CD8^+^ T-cell numbers, but also leads to the decrease of CD44^+^CD8^+^ effector/memory T cells in steady-state. These observations prompted us to explore Brd4 function in CD8^+^ T-cell response to infection. To determine the role of *Brd4* in regulation of CD8^+^ T cells response to viral infection, we next established an acute viral infection mouse model to examine the differentiation and function of CD8^+^ T cells upon *Brd4* deletion. Due to the decreased number of T cells in *Brd4* ^-/-^ mice, a co-adoptive transfer strategy was used to overcome this imbalance. Equal number of congenially marked *Brd4^-/-^* and *Brd4^+/+^* P14^+^CD8^+^ T cells, which specifically recognize the LCMV-gp33 epitope presented by H2-D^b^, were mixed and transferred into same wild-type recipient mice to explore the role of Brd4 in CD8^+^ T-cell clonal expansion and differentiation. One day later, recipient mice were infected with the Armstrong strain of LCMV to initiate an acute viral infection (**Figure 3A**). The number of *Brd4* ^-/-^ effector P14^+^CD8^+^ T cells recovered from the spleen and lymph nodes was significantly decreased to approximately 10% of *Brd4* ^+/+^ effector P14^+^CD8^+^ T cells at day 8 (**Figure 3B**). In addition to the lymphoid organs, a consistent reduction of *Brd4* ^-/-^ effector P14^+^CD8^+^ T cells in peripheral blood and liver were observed when compared to their counterpart (**Figure 3B**). Moreover, there was an approximately 5-fold reduction of memory P14^+^CD8^+^ T cells at day 35 post-infection upon *Brd4* deletion (**Figure 3C**). The reduction of memory P14^+^CD8^+^ T cells upon *Brd4* deletion largely results from defective clonal expansion of effector P14^+^CD8^+^ T cells, which differentiate into memory cells after the resolution of infection.

The differential expression of CD127 and KLRG1 distinguishes the terminal differentiation subset (KLRG1^+^CD127^-^), which fades *via* apoptosis, from memory precursors (KLRG1^-^CD127^+^), which survives to develop into memory cells within effector cells. To further examine whether *Brd4* deficiency affects effector P14^+^CD8^+^ T cells populations, the composition of effector P14^+^CD8^+^ T cells was analyzed by the expression of CD127 and KLRG1. When 5000 *Brd4^+/+^* and *Brd4^-/-^* naïve P14^+^CD8^+^ T cells were co-adoptively transferred into recipient mice, we found that Brd4 deletion barely affected effector CD8^+^ T cells differentiation (**Supplemental Figure 3A**). As the number of transferred P14 cells can affect the outcome of LCMV infection (40), we checked whether Brd4 deletion could influence the differentiation of CD8^+^ T cells if a physiological number of P14 cells (~1000 cells) were transferred into recipient. Unexpectedly, the loss of *Brd4* significantly impaired the differentiation of KLRG1^+^CD127^-^ terminally differentiated subset, and enhanced the differentiation of KLRG1^-^CD127^+^ memory precursors (**Supplemental Figure 3B**). However, *Brd4* deficiency barely affected the cytotoxicity of effector P14^+^CD8^+^ T cells evidenced by equal *in vivo* killing activity and production of granzyme B and CD107a (**Supplemental Figures 3C–E**). In addition, cytokines secretion including IFN-γ and IL-2 by *ex vivo* peptide stimulation suggested that *Brd4* deletion had no effect on proinflammatory cytokine production (**Supplemental Figure 3F**). Together, these findings demonstrate that *Brd4* is required for effector CD8^+^ T-cell clonal expansion and terminal differentiation during viral infection.

### Brd4 Regulates Glucose and Migration-Related Genes Transcription in CD8^+^ T Cells

**Figure 4:**
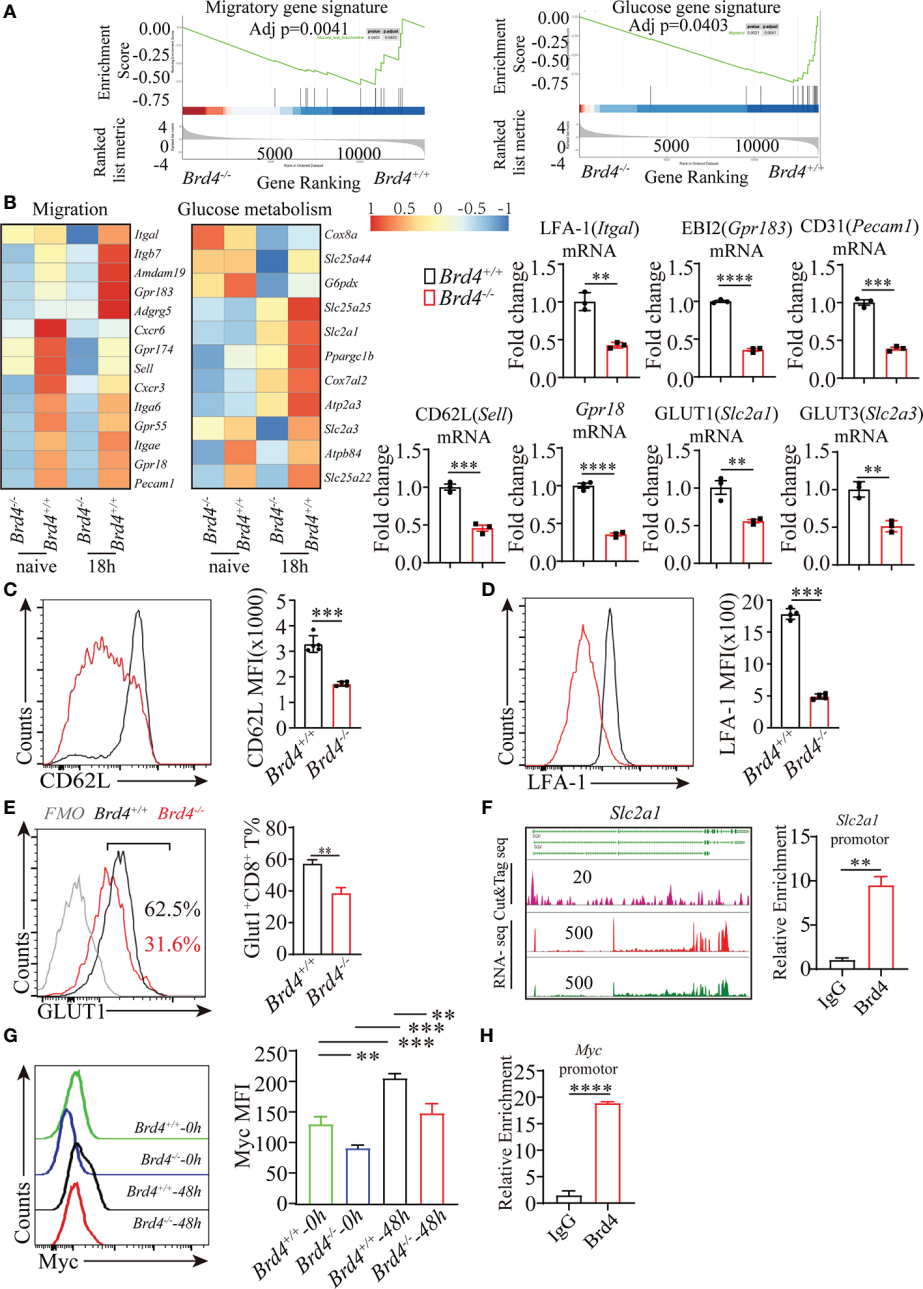
Brd4 regulates migration- and glucose metabolism-related gene transcription. **(A)** GSEA enrichment analysis of genes involved in glucose metabolism and cell migration. **(B)** Heat map showing the downregulated genes in CD8^+^ T cells after Brd4 deletion and RT-qPCR confirmation of downregulated genes. **(C)** Flow cytometry showing the expression of CD62L in naïve CD8^+^ T cells. **(D)** Flow cytometry showing the expression of LFA-1 in naïve CD8^+^ T cells. **(E)** Flow cytometry analysis of GLUT1 expression in naïve CD8^+^ T cells. **(F)** CUT&Tag analysis showing the binding sites of BRD4 in Slc2a1 gene loci (left) and ChIP-qPCR confirmation of the enrichment of BRD4 in Slc2a1 loci in CD8^+^ T cells (right). **(G)** Intracellular staining for Myc detection in Brd4^+/+^ and Brd4^-/-^ naïve and activated CD8^+^ T cells. **(H)** ChIP-qPCR confirmation of the enrichment of BRD4 in Myc locus in CD8^+^ T cells. The data was analyzed by two-tailed unpaired *t*-test between two independent groups. Results were indicated as mean ± sem (error bars). **p < 0.01; ***p < 0.001; ****p < 0.0001. Each experiment repeats more than 3 times.

To uncover the underlying mechanism responsible for disrupted homeostasis and defective immune response of *Brd4^-/-^* CD8^+^ T cells, we performed transcriptome analysis with naïve and in-vitro activated CD8^+^ T cells. Compared to *Brd4^+/+^* naïve CD8^+^ T cells, *Brd4* depletion led to 956 differentially downregulated genes and 841 differentially upregulated genes (**Supplemental Figure 4A**). Moreover, the downregulated genes exceeding upregulated genes are more evident in activated *Brd4^-/-^* CD8^+^ T cells, indicating that Brd4 is highly involved in CD8^+^ T-cell activation (**Supplemental Figure 4A**). Further GSEA enrichment analysis revealed that genes associated with glucose metabolism were downregulated in the *Brd4 ^-/^* ^-^ CD8^+^ T cells (**Figure 4A**). Also, migration-related genes were downregulated in the *Brd4 ^-/^* ^-^ CD8^+^ T cells (**Figure 4A**).

Among these differentially downregulated genes, several genes including *Sell*, *Itgal*, *Gpr183*, *Gpr18*, *Pecam1*, *Slc2a1*, and *Slc2a3* were selected for further confirmation. RT-qPCR analysis showed consistent downregulation of these selected genes in *Brd4^-/-^* CD8^+^ T cells in comparison to their counterparts (**Figure 4B**). In addition, the expression of CD62L (*Sell*), LFA-1 (*Itgal*), and GLUT1 (*Slc2a1*) was also decreased in *Brd4^-/-^* CD8^+^ T cells (**Figures 4C–E**). To determine whether the transcription repression of these selected genes was regulated by Brd4 directly or indirectly, we generated a CUT&Tag library to profile the binding sites of Brd4 throughout the genome in naïve CD8^+^ T cells. By combining the RNA-seq signal with the CUT&Tag signal, we observed that Brd4 seemed to regulate the transcription of these migratory genes in an indirect fashion in naïve CD8^+^ T cells, as no obvious CUT&Tag signal was detected at these gene loci except for *Gpr183* (**Supplemental Figure 4B**). Whether Brd4 could directly regulate the transcription of these migratory gene in other CD8^+^ T cell subsets remain to be defined, due to the dynamic occupancy of Brd4 throughout the genome and chromatin remodeling in the course of CD8^+^ T cells differentiation. However, Brd4 directly bound to the *Slc2a1* gene locus and the transcription of *Slc2a1*(GLUT1) was inhibited upon *Brd4* deletion (**Figure 4F**). Further chromatin immunoprecipitation, followed by qPCR analysis, confirmed that Brd4 was highly enriched in the promotor region of *Slc2a1* (GLUT1), suggesting that Brd4 directly regulates *Slc2a1* transcription in CD8^+^ T cells (**Figure 4F**).

Myc promotes tumor growth partially through increasing glycolysis, including elevated glucose uptake, by facilitating glucose transporter GLUT1 expression. Because targeting Brd4 slows down tumor growth partially through downregulating Myc expression, we then investigated whether the downregulation of glucose transporter GLUT1 resulted from the reduced Myc expression in *Brd4^-/-^* CD8^+^ T cells (30, 41). The intracellular staining for Myc measurement indicated that Myc was highly induced when naïve *Brd4^+/+^* CD8^+^ T cells received TCR stimulation, which was consistent with TCR stimulation quickly inducing Myc expression in T cells (18). However, activated *Brd4^-/-^* CD8^+^ T cells failed to efficiently induce Myc expression in comparison to *Brd4^+/+^* CD8^+^ T cells (**Figure 4G**). Moreover, we verified that Brd4 highly enriched in Myc locus (**Figure 4H**). These observations indicate that Brd4 regulates CD8^+^ T-cell glucose metabolism not only through directly regulating GLUT1 expression, but also indirectly regulating Myc expression.

### *Brd4* Maintains CD8^+^ T-Cell Homeostasis by Promoting Homing and Glucose Metabolism in Mitochondria

**Figure 5:**
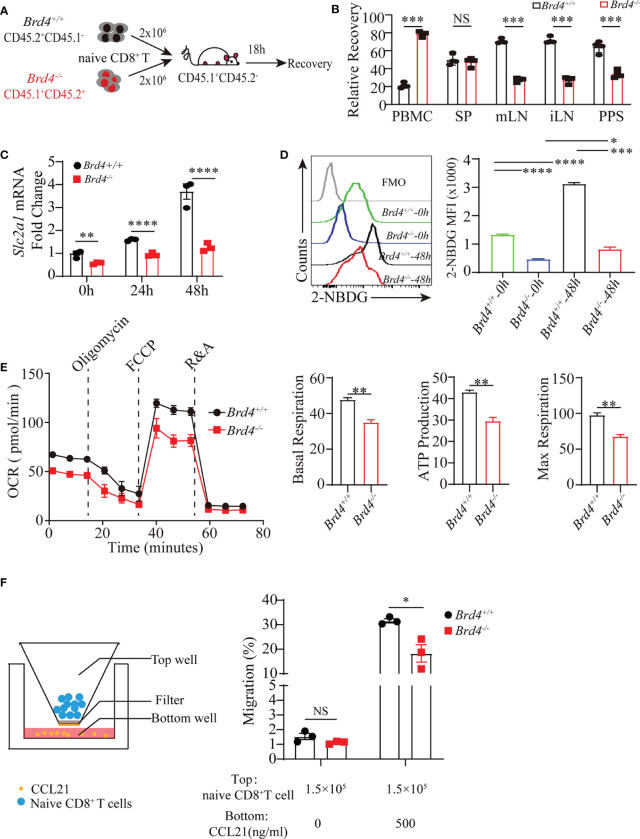
Brd4 ensures CD8^+^ T cells homing and mitochondrial glucose oxidation. **(A)** Experiment setup. Purified *Brd4* ^+/+^ (CD45.1^-^CD45.2^+^) and *Brd4* ^-/-^ (CD45.1^+^CD45.2^+^) naïve CD8^+^ T cells were mixed at a 1:1 ratio and then transferred into CD45.1^+^CD45.2^-^ recipient mice. Donor cell recovery was checked in indicated tissues to assess their homing ability after 18h post-transfer. **(B)** Relative recovery of donor naïve CD8^+^ T cells in all checked organs. **(C)** RT-qPCR analysis of *Slc2a1* transcription at distinct time point after CD8^+^ T cell receiving TCR stimulation. **(D)** Glucose uptake measurement in naïve and *in vitro*-activated CD8^+^ T cells from *Brd4^+/+^* and *Brd4^-/-^* mice. **(E)** Oxidative phosphorylation in *Brd4^+/+^* and *Brd4^-/-^* naïve CD8^+^ T cells was determined by measuring oxygen consuming rate after adding indicated compounds. **(F)** Schematic of *in vitro* migration assay in transwell plate (left) and the migration efficiency of *Brd4^+/+^* and *Brd4^-/-^* naïve CD8^+^ T cells in response to CCL21 chemotaxis (right). Two-tailed unpaired *t*-test was used to analyze two independent groups, while paired Student’s *t*-test was used when sample being compared from same mouse. To compare multiple groups, we used an analysis of variance (ANOVA). Results were indicated as mean ± sem (error bars). *p < 0.05; **p < 0.01; ***p < 0.001; ****p < 0.0001. NS, not significant. Each group includes at least 3 mice and each experiment repeats more than 2 times.

Recirculation is crucial for maintaining T-cell homeostasis. Naïve T cells greatly rely on homing receptors (CCR7, CD62L and LFA-1) to support their migration into lymph nodes to access to the stimulation of homeostatic cues, which mediates naïve T-cell survival and homeostatic proliferation. To functionally test whether naïve CD8^+^ T cells from *Brd4^-/-^* mice fail to efficiently migrate into lymph nodes due to the downregulation of CD62L and LFA-1, equal number of *Brd4^-/-^* and *Brd4^+/+^* naïve CD8^+^ T cells were mixed at a 1:1 ratio and then transferred into same recipient mice. The ability to migrate into lymph nodes was determined at 18h post-transfer (**Figure 5A**). Compared to *Brd4^+/+^* naïve CD8^+^ T cells, there were fewer *Brd4^-/-^* naïve CD8^+^ T cells capable of migrating into lymph nodes, but more *Brd4^-/-^* naïve CD8^+^ T cells were trapped in peripheral blood (**Figure 5B**). In addition, we shortened the sample-harvesting time to 4 h post-transfer in the homing assay to exclude the potential side-effect of a longer time and observed a similar result (**Supplemental Figures 5A, B**). Because homing receptors CD62L and LFA-1 are not required for naïve T cells to traffic into spleen, a comparable recovery was observed in spleen post-transfer (**Figure 5B**). In addition, marked downregulation of CD62L and LFA-1 in *Brd4^-/-^* naïve CD8^+^ T cells from peripheral blood was observed, but not for CCR7, which is consistent with defective homing (**Supplemental Figures 5C–E**). These results suggest that Brd4 controls naïve CD8^+^ T-cell homing by regulating CD62L and LFA-1 expression. Impaired trafficking into lymph nodes results in an increased accumulation of T cells in the blood and spleen. However, *Brd4^-/-^* mice also have decreased T-cell numbers in spleen and peripheral blood, indicating defective homing could not fully explain the reduction of T cells. Thus, impaired trafficking synergizing with decreased survival capability could simultaneously contribute to the reduced pool of CD8^+^ T cells.

The above observations suggest that *Brd4* deficiency downregulates glucose transporter GLUT1 expression in naïve CD8^+^ T cells. As naïve CD8^+^ T cells largely depend on glucose uptake to undergo oxidative phosphorylation, which supports naïve T-cell survival, we then explored whether *Brd4* deficiency could cause impaired cell survival by decreasing glucose metabolism (10, 13). Naïve T cells increased glucose uptake by upregulating GLUT1 expression upon activation, while there was an approximately 2-fold reduction of GLUT1 transcription in *Brd4* ^-/-^ CD8^+^ T cells after TCR stimulation (**Figure 5C**). Moreover, glucose uptake was directly measured by evaluating 2-NDBG (2-deoxy-2-[(7-nitro-2,1,3-benzoxadiazol-4-yl) amino]-D-glucose) fluorescent signal after incubating CD8^+^ T cells with 2-NBDG. Approximately 50% and 70% reduction of glucose uptake in *Brd4^-/-^* naïve and activated CD8^+^ T cells were observed when compared to the *Brd4^+/+^* counterpart, respectively (**Figure 5D**). We next checked whether the decreased glucose input affected oxidative phosphorylation. Oxidative phosphorylation analysis determined by oxygen consuming rate indicated that *Brd4^-/-^* naïve CD8^+^ T cells markedly decreased oxidative phosphorylation relative to their counterpart (**Figure 5E**). Energy production in mitochondria not only maintains naïve CD8^+^ T cells survival, but also supports cell migration. Then, we examined whether *Brd4* ^-/-^ naïve CD8^+^ T cells exhibited defective *in vitro* migration. Migration analysis showed that *Brd4* ^-/-^ naïve CD8^+^ T cells had reduced capability of migration responding to CCL21 chemotaxis (**Figure 5F**). Together, these data demonstrate that Brd4 is important for naïve CD8^+^ T-cell survival and migration by facilitating adequate glucose uptake to fuel oxidative phosphorylation in mitochondria.

### Brd4 Supports CD8^+^ T-Cell Proliferation by Inducing Glycolysis After Activation

**Figure 6:**
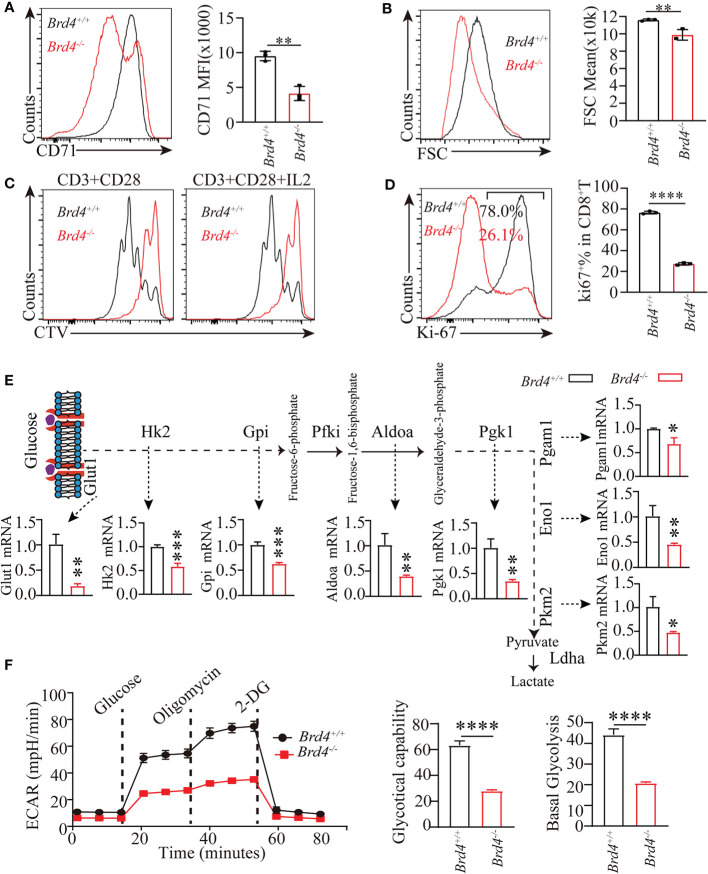
Brd4 promotes CD8^+^ T cell proliferation through inducing glycolysis. **(A)** Purified *Brd4^+/+^* and *Brd4^-/-^* naïve CD8^+^ T cells were activated with anti-CD3/28 antibody for 24 hours to assess cell activation. Flow cytometry showing the expression of CD71 after stimulation (left) and the quantification of CD71 MFI (right). **(B)** Flow analysis of cell size of *in vitro*-activated CD8^+^ T cells. **(C)** Purified *Brd4^+/+^* and *Brd4^-/-^* naïve CD8^+^ T cells were labeled with CellTrace Violet (CTV) and activated with anti-CD3/28 antibody in the presence or absence of IL-2 for 3 days to assess cell proliferation. Flow cytometry showing CTV dilution. **(D)** Intracellular staining showing the expression of Ki-67 in *Brd4^+/+^* and *Brd4^-/-^* CD8^+^ T cells after activation for 24h (left) and the quantification of Ki-67^+^CD8^+^ T cells (right). **(E)** qPCR analysis showing the differentially expressed glycolytic genes in *Brd4^+/+^* and *Brd4^-/-^* CD8^+^ T cells. **(F)** Chart showing glycolysis measurement of *in vitro*-activated CD8^+^ T cells (left) and the quantification of the basal and maximal glycolysis (right). Two-tailed unpaired *t*-test was used to analyze two independent groups. Results were indicated as mean ± sem (error bars). *p < 0.05; **p < 0.01; ***p < 0.001; ****p < 0.0001. Each group includes at least 3 mice and each experiment repeats more than 2 times.

Having established the critical role of *Brd4* in regulation of glucose uptake which plays an essential function in regulating CD8^+^ T-cell response, we further investigated whether the defective clonal expansion of CD8^+^ T cells resulted from deregulated glucose metabolism. To this end, naïve CD8^+^ T cells isolated from *Brd4^-/-^* and *Brd4^+/+^* mice were activated by anti-CD3 and anti-CD28 antibodies. Compared to the *Brd4^+/+^* CD8^+^ T cells, activated *Brd4^-/-^* CD8^+^ T cells markedly downregulated the expression of CD71, an activation marker (**Figure 6A**). Moreover, activated CD8^+^ T cells underwent growth to an enlarged cell size, while *Brd4^-/-^* CD8^+^ T cells failed to efficiently enlarge their size after stimulation (**Figure 6B**). Consistent with the impaired activation, *Brd4^-/-^* CD8^+^ T cells had undergone 1~2 rounds division after stimulation for 3 days *in vitro*, while almost all *Brd4^+/+^* CD8^+^ T cells had undergone cell division up to five times (**Figure 6C**). Moreover, there was an approximately 70% reduction of Ki-67^+^CD8^+^ T cells within activated *Brd4^-/-^* CD8^+^ T cells in comparison to *Brd4^+/+^* CD8^+^ T cells (**Figure 6D**). An elevated level of apoptosis was observed in activated CD8^+^ T cells after *Brd4* deletion (**Supplemental Figures 6A**). The treatment of CD8^+^ T cells with JQ1, a pan-BRD inhibitor that blocks *Brd4* function, also led to a similar phenotype as *Brd4* deletion (**Supplemental Figures 6B–E**). These data indicate that *Brd4* supports CD8^+^ T-cell activation, proliferation, and survival after receiving TCR stimulation *in vitro*.

Previous studies shows that naïve CD8^+^ T cells induce glycolysis to support cell growth, full activation, and proliferation after TCR signal stimulation. Glycolysis largely depends on the induction of transcription factor Myc expression and elevated glucose uptake (10, 18). Our findings suggest that *Brd4^-/-^* CD8^+^ T cells decrease Myc expression and glucose uptake after TCR signal stimulation. To explore whether an impaired *Brd4* ^-/-^ CD8^+^ T-cell response results from defective glycolysis, we measured glycolytic capability *in vitro* with activated *Brd4^-/-^* and *Brd4^+/+^* CD8^+^ T cells. Firstly, the expression of genes involved in glycolysis were checked in activated CD8^+^ T cells. Transcription analysis revealed that *Brd4^-/-^* CD8^+^ T cells downregulated the transcription of many glycolytic genes in comparison to *Brd4^+/^* ^+^ CD8^+^ T cells after activation (**Figure 6E**). Furthermore, glycolytic flux analysis by detecting extracellular acid rates showed that activated *Brd4^-/-^* CD8^+^ T cells severely decreased glycolysis relative to activated *Brd4^+/+^* CD8^+^ T cells after sequential addition of compounds including glucose, oligomycin, and 2-DG (2-Deoxy-D-glucose) (**Figure 6F**).

Our above data indicate that *Brd4* deficiency downregulates LFA-1 expression in naïve CD8^+^ T cells. It has been shown that LFA-1 also gets involved in regulating T cell activation and proliferation (42–45). To rule out the potential possibility that defective proliferation of *Brd4^-/-^* CD8^+^ T cells results from the downregulation of LFA-1, we evaluated the effect of LFA-1 knockdown on CD8^+^ T proliferation and survival. Three distinct shRNAs targeting LFA-1 were designed to downregulate LFA-1 in CD8^+^ T cells and all of them could mediate approximately 50% knockdown efficiency (**Supplemental Figures 7A, B**). In contrast to *Brd4^+/+^* CD8^+^ T cells, *Brd4^-/-^* CD8^+^ T cells undergone limited proliferation after activation *in vitro*, while *Brd4^+/+^* CD8^+^ T cells transduced with shLFA-1 exhibited similar proliferation pattern to shNC control, suggesting that LFA-1 knockdown barely decreased CD8^+^ T cells proliferation after *in vitro* activation with soluble anti-CD3 and CD28 antibodies (**Supplemental Figures 7C, D**). Also, LFA-1 knockdown seemed not to impair CD8^+^ T cells survival (**Supplemental Figures 7E**). The bare effect of LFA-1 knockdown on CD8^+^ T cells proliferation after *in vitro* activation with soluble anti-CD3 and CD28 antibodies emphasizes that the mechanism of LFA-1 promoting proliferation is through stabilizing interaction between T cells and antigen-presenting cells, which is consistent with the previous report (43). As LFA-1 gets involved in regulating T cells proliferation by stabilizing the interaction between antigen-presenting cells and T cells, we tried to examine whether the reduced expression of LFA-1 in naïve CD8^+^ T cells resulted in defective clonal expansion of effector P14^+^CD8^+^ T cells *in vivo*. To this end, we firstly analyzed the dynamic expression of LFA-1 in *Brd4* ^+/+^ and *Brd4* ^-/-^ naïve CD8^+^ T cells after *in vitro* activation, as TCR signal stimulation upregulates LFA-1 expression. The dynamic expression of LFA-1 showed that the TCR activation strongly induced the expression of LFA-1 in both *Brd4* ^+/+^ and *Brd4* ^-/-^ CD8^+^ T cells at indicated time points and the gap of LFA-1 expression between naïve *Brd4* ^+/+^ and *Brd4* ^-/-^ CD8^+^ T cells was gradually narrowed down after activation (**Supplemental Figure 7F**). Besides, we observed that the shNC-transduced *Brd4^-/-^* CD8^+^ T cells expressed a similar level of LFA-1 as that in shNC-transduced *Brd4^+/+^* CD8^+^ T cells (**Supplemental Figure 7G**). Meanwhile, the expression of LFA-1 in *Brd4^-/-^* and *Brd4^+/+^*effector P14^+^CD8^+^ T cells recovered from same acutely infected host was firstly evaluated with flow cytometry. Unexpectedly, although *Brd4^-/-^* P14^+^CD8^+^ T cells exhibited impaired clonal expansion relative to their counterparts, comparable LFA-1 expression was detected in both P14^+^CD8^+^ T cells (**Supplemental Figure 7H**). Because TCR signal stimulation increases the surface amount of LFA-1 in CD8^+^ T cells, we re-analyzed the LFA-1 expression in CD44^-^CD8^+^ and CD44^+^CD8^+^ T cells from *Brd4^+/+^* and *Brd4^-/-^* mice. Expression analysis indicated that *Brd4* deficiency only downregulated LFA-1 in naïve CD44^-^CD8^+^ T cells rather in activated CD44^+^CD8^+^ T cells (**Supplemental Figure 7I**). As *Brd4* deletion barely downregulates LFA-1 expression in activated CD8^+^ T cells, we excluded the possibility that the impaired *in vivo* clonal expansion of *Brd4^-/-^* P14*^+^*CD8^+^ T cells resulted from the defective immune synapse formation. Together, these observations suggest that Brd4 promotes proliferation through inducing Myc expression and glucose uptake, both of which facilitate glycolysis induction in CD8^+^ T cells after TCR stimulation.

### Brd4 Inhibition Dampens CD8^+^ T Cell-Mediated Adaptive Immunity During Acute Viral Infection

**Figure 7:**
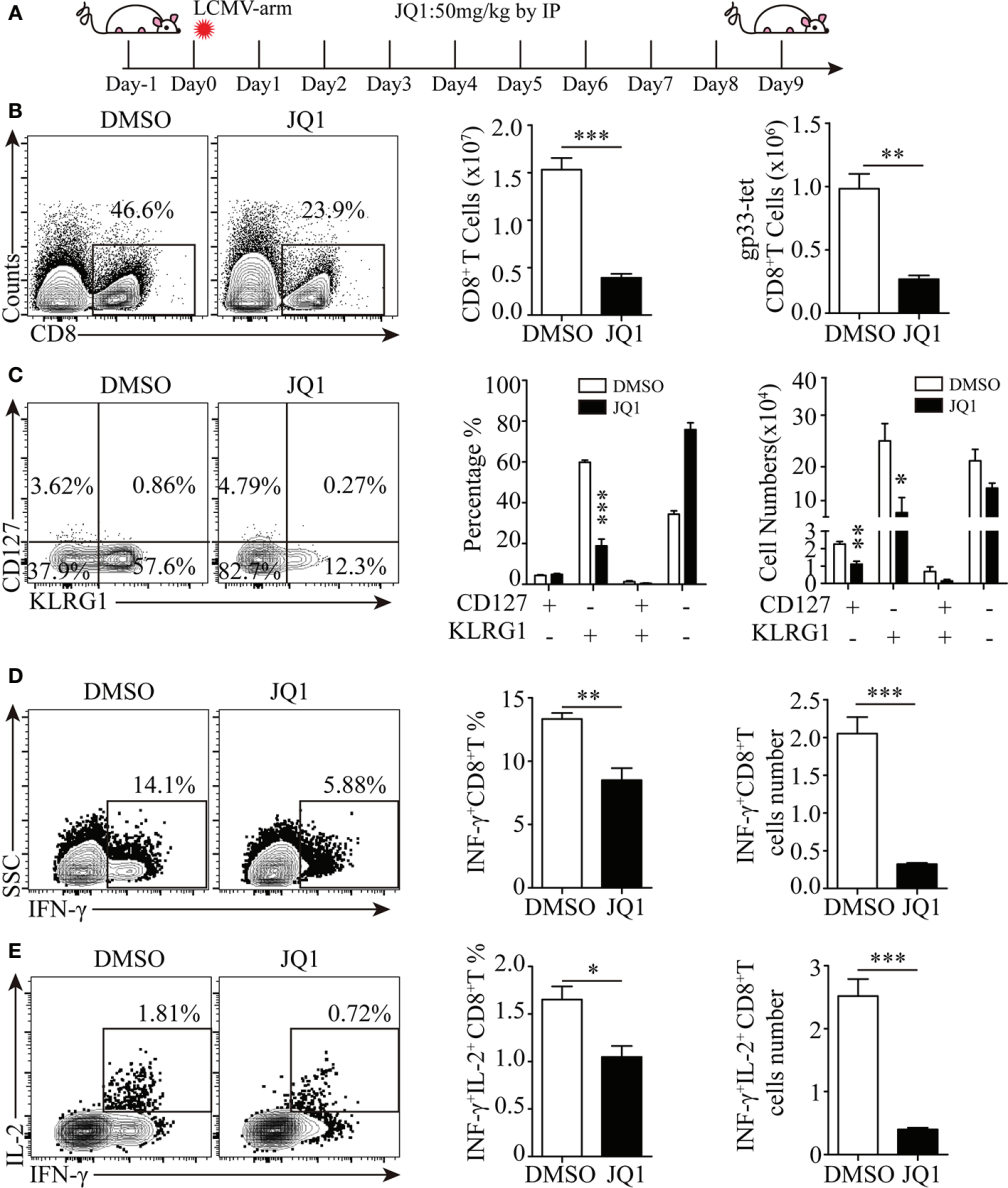
Targeting Brd4 with inhibitors dampens effector CD8^+^ T cells-mediated antiviral immunity. **(A)** Experiment design. **(B)** Flow cytometry showing the expression of CD8 (left), the quantification of total CD8^+^ T cells (middle) and the quantification of LCMVgp33 epitope-specific CD8^+^ T cell numbers (right). **(C)** Flow cytometry showing the expression of KLRG1 and CD127 in LCMVgp33 epitope-specific CD8^+^ T cells from mice treated with JQ1 or DMSO at day 8 post-infection (left) and the frequency and number of each subset (right). **(D, E)** Flow cytometry analysis of indicated cytokines production after peptide stimulation in CD8^+^ T cells from mice treated with JQ1 or DMSO (left) and the number and frequency (right) of cytokine-producing CD8^+^ T cells. The data were analyzed by two-tailed unpaired *t*-test between two independent groups. Results were indicated as mean ± sem (error bars). *p < 0.05; **p < 0.01; ***p < 0.001. Each group includes at least 4 mice and each experiment repeats more than 2 times.

As Brd4 inhibition with small molecules has attracted great attention in cancer and viral infection therapies, we then evaluated whether Brd4 inhibition represses the CD8^+^ T-cell response to viral infection. To this end, an acute LCMV infection mouse model was established to study the effect of Brd4 inhibition with JQ1, a pan-BRD inhibitor which blocks Brd4 proteins from binding to acetylated histones, on endogenous antigen-specific CD8^+^ T-cell function and differentiation. JQ1 was administrated daily *via* intraperitoneal injection into acutely infected wild-type mice (**Figure 7A**). We then evaluated the function and differentiation of endogenous antigen-specific CD8^+^ T cells at the peak of the immune response. There was approximately 3-fold reduction of total and LCMVgp33-specific CD8^+^ T cells from mice receiving JQ1 treatment (**Figure 7B**). Furthermore, LCMVgp33-specific CD8^+^ T cells from JQ1-treated mice displayed a severe defect in terminal differentiation, as the KLRG1^+^CD127^-^ population was significantly decreased both in frequency and number (**Figure 7C**). In addition, there was a functional impairment in the effector CD8^+^ T cells from JQ1-treated mice that is manifested by less IFN-γ and IL-2 secretion after *ex vivo* stimulation (**Figures 7D, E**). Although the phenotype of antigen-specific CD8^+^ T cells in JQ1-treated mice is similar to the phenotype of *Brd4^-/-^* P14^+^CD8^+^ T cells, BET inhibition has more severe defect in terminal differentiation and cytokine production. This discrepancy probably results from the potential compensative effect of Brd2 or Brd3 in CD8^+^ T cells after *Brd4* deletion (23). Also, it is important to note that BET inhibition impairs CD8^+^ T cell differentiation through directly inhibiting the function of Brd4 in other cell types, such as DC and Macrophage. Collectively, these results indicate that targeting Brd4 with small molecules severely dampens adaptive immunity by restricting antigen-specific CD8^+^ T-cell clonal expansion and effector differentiation.

## Discussion

**Figure 8:**
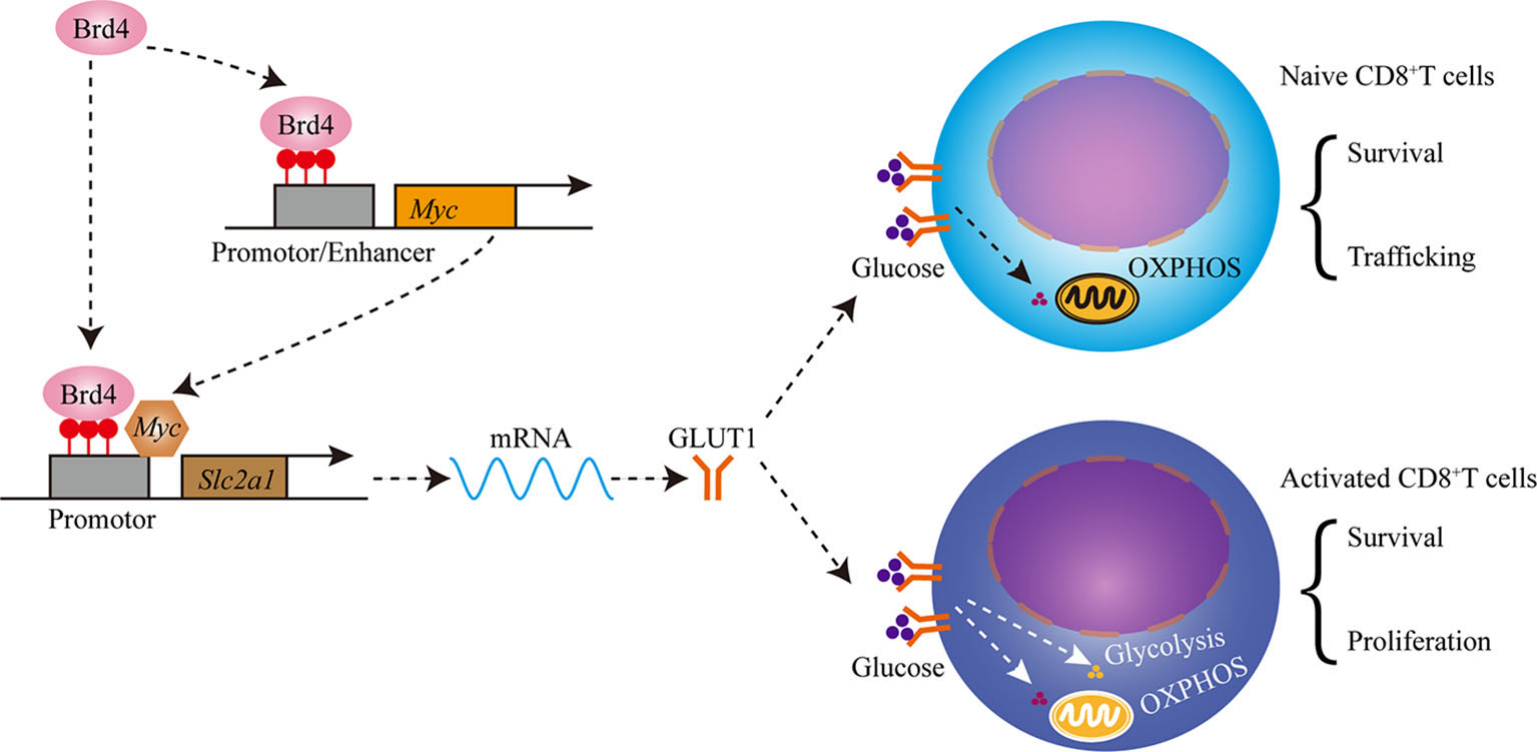
Schematics showing that Brd4 regulates CD8^+^ T cell homeostasis and proliferation through controlling glucose metabolism. Brd4 maintains naïve CD8^+^ T cells homeostasis by regulating the survival and trafficking of naïve CD8^+^ T cells in steady-state. Brd4 promotes efficient glucose uptake by upregulating GLUT1 (glucose transporter 1) and Myc expression, which glucose oxidation in mitochondria fuels naïve CD8^+^ T cells survival and trafficking. Upon activation, activated CD8^+^ T cells increase glucose uptake to meet the bioenergetic and biosynthetic demands, which includes quick proliferation, survival and effector differentiation.

CD8^+^ T cells play a central role in the containment of infectious agents and tumor. Normal homeostasis maintenance, targeted migration, efficient proliferation, and functional differentiation are required to mount a robust immune response. Therefore, the identification of regulators responsible for these processes is critical for devising immunomodulatory or immunotherapeutic strategies. In this study, we found that Brd4 regulated CD8^+^ T-cell homeostasis and immunity *via* controlling glucose metabolism and trafficking. Brd4 maintained naïve CD8^+^ T-cell homeostasis by indirectly controlling the expression of homing receptors which are required for efficient trafficking. In addition, Brd4 promoted glucose uptake to generate adequate ATP levels to support naïve CD8^+^ T-cell survival by enhancing Myc and GLUT1 expression. Upon activation, Brd4 facilitated glycolysis induction to support the quick proliferation and efficient effector differentiation of activated CD8^+^ T cells by enhancing glucose uptake. Furthermore, targeting Brd4 with JQ1, a pan-BRD inhibitor, impeded CD8^+^ T-cell clonal expansion and effector differentiation during acute viral infection. Thus, our findings reveal a previously-unappreciated role of Brd4 in the regulation of peripheral naïve CD8^+^ T-cell homeostasis and immunity through controlling glucose metabolism (**Figure 8**).

Importantly, in consistent with our finding, a recent study also supports the importance of Brd4 in CD8^+^ T cells differentiation, in which Brd4 bound diverse regulatory regions of effector-related genes to enforce terminal differentiation of CD8^+^ T cells in the setting of viral infection and tumorigenesis (46). However, our study proved the indispensable role of Brd4 in CD8^+^ T cells from different perspectives: (1) Brd4 regulates naïve CD8^+^ T cells homeostasis by controlling naïve CD8^+^ T cells survival and trafficking, as *Brd4* deficiency results in remarkable loss of CD8^+^ T cells and altered CD8^+^ T cells population in the steady-state; (2) Brd4 establishes a novel link between epigenetic regulation and immune metabolism by controlling GLUT1 expression; (3) Brd4 inhibition raises the concern/potential risk of immunosuppressive effect in viral infection therapy, such as HIV-1 latency, based on the critical role of Brd4 in promoting the clonal expansion and effector differentiation of CD8^+^ T cells *in vivo*.

To efficiently accommodate bioenergetic and biosynthetic demands, effector CD8^+^ T cells preferentially utilize glycolysis instead of oxidative phosphorylation (10, 11, 47). Although glycolysis produces less ATP than oxidative phosphorylation, glycolysis provides sufficient substrate for biomacromolecule synthesis, which is essential for CD8^+^ T-cell proliferation and effector differentiation (10, 11, 18). We found that *Brd4* deletion profoundly impaired the proliferation and effector differentiation of CD8^+^ T cells by limiting glucose uptake. Decreased glycolytic flux further represses CD8^+^ T-cell activation, growth, survival, and proliferation by limiting the availability of building blocks. In addition, constitutive glycolytic metabolism promotes effector-memory CD8^+^ T-cell differentiation during viral infection (48). Furthermore, *Brd4* ^-/-^ mice harbor less CD44^+^CD8^+^ effector-memory T cells, which is partially in line with the defective glycolytic flux. However, whether the altered glucose metabolism in *Brd4^-/-^* CD8^+^ T cells influences memory CD8^+^ T-cell differentiation needs to be defined in future studies.

Tumor cells could evade immune destruction by competing with T cells for glucose in tumor microenvironment. The glucose-poor microenvironment profoundly limits T-cell effector response to tumor through decreasing IFN-γ expression (49–51). We observed that *Brd4* deletion or inhibition profoundly impairs the clonal expansion and effector differentiation during acute infection due to the reduced glucose uptake. Moreover, antigen-specific CD8^+^ T cells from JQ1-treated mice exhibits decreased IFN-γ secretion. Recently, emerging evidence demonstrates that targeting Brd4 with small molecules shows great potential for cancer cell elimination (52). Baded on our findings, Brd4 inhibition could erode antitumor immunity of CD8^+^ T cells by limiting proliferation and effector differentiation. Thus, when targeting Brd4 with inhibitors, its potential inhibitory effect on CD8^+^ T cells differentiation and function should be taken into consideration. Considering the inhibitory effect of Brd4 inhibition on CD8^+^ T cells function and differentiation, it could not be feasible to combine Brd4-targeting therapy with CAR-T-/TCR-T-based immunotherapy for incurable cancer. Also, we speculate that targeting Brd4 with inhibitors is more likely to eliminate tumor cells in “cold tumor” defined as the lack of immune cell infiltration, while “hot tumor,” defined as with relative abundant immune cells infiltration may not respond well to Brd4 inhibition, as targeting Brd4 simultaneously acts on tumor cells and CD8^+^ T cells.

Although great achievements have been made in human immunodeficiency virus type 1 (HIV-1) infection therapy, viremia quickly rebounds after the cessation of combined antiretroviral therapy (cART) due to HIV-1 latency infection. Thus, HIV-1 latency infection represents a major barrier for pure elimination of HIV-1 infection. Current approaches to HIV-1 infection therapy focus on “shock” and “kill” (53). In this therapy, latency reverse agents are used to reactivate latent HIV-1 proviruses, and then host killer cells including but not limited to CD8^+^ T-cells, destruct virus-producing target cells to purge HIV-1 reservoirs (53). The epigenetic reader Brd4 facilitates HIV-1 latency maintenance by competing against the pTEF-b complex with viral protein Tat or recruiting repressive SWI/SNF chromatin remodeling complex to inhibits HIV-1 transcription (33–36). Manipulation of Brd4 with RNAi-mediated knockdown or inhibitors awaken the latent HIV-1 proviruses (33–36, 54–57). Thus, Brd4 is a promising candidate for latency reverse agent development. However, the effect of *Brd4* inhibition on CD8^+^ T cells function and differentiation remains unclear. Deep understanding the potential effects of Brd4 inhibition accelerates the progress of clinical trial. Our observations demonstrate that treatment of CD8^+^ T cells with JQ1, a pan-BRD inhibitor, severely restricts the clonal expansion and effector differentiation of CD8^+^ T cells *in vivo*. In addition, inhibition of *Brd4* with small molecules profoundly impairs human CD4^+^ and CD8^+^ T-cell function *in vitro (* 58, 59). Although small molecules targeting Brd4 could potently reactivate HIV-1 latency, CD8^+^ T cells in patient with HIV-1 would fail to execute the function of immune-destruction due to the inhibitory effect of Brd4. Therefore, Brd4 inhibitor used as HIV-1 latency reverse agent for antiviral therapy should be more cautious.

Transcriptome analysis identified that *Brd4* depletion led to the downregulation of many genes associated with migration, including but not limited to CD62L, LFA-1, CXCR6, and CD103. The mechanism by which *Brd4* deletion downregulates the expression of these trafficking molecules remains unknown. Indeed, LFA-1, CXCR6, and CD103(*Itage*) are crucial for CD8^+^ tissue resident memory (Trm) T cell formation and positioning, indicating that Brd4 potentially regulates Trm formation and function (60, 61). Importantly, CD103^+^ Trm in tumors is positively correlated with a better prognosis, and CD103 or LFA-1 engagement between cytotoxic T cells and tumor cells augments effector function (62, 63). Currently, more attention is being paid to the effect of Brd4 inhibition on tumor cell growth, while its effect on the function and differentiation of intratumor CD8^+^ T cells is spare. It is worth exploring the role of Brd4 in the differentiation of intratumor CD103^+^CD8^+^Trm in further study.

Recently, targeting Brd4 with inhibitors has received considerable attraction and is broadly applied to a wide spectrum of disease from ongoing clinical trial on cancer to cardiovascular disease (31, 32, 64). The prominent side-effect of *Brd4* inhibition is memory loss in mice treated with pan-BRD inhibitor JQ1 and hearing loss in mice with specific deletion of *Brd4* in hair cells so far (65, 66). However, our study established a critical role of Brd4 in regulating CD8^+^ T cells antiviral immunity. These observations hold significant clinical implication for ongoing clinical trials. Patients receiving Brd4 inhibitors could cause attenuated antiviral immunity, due to the potential defective CD8^+^ T cells response to infection. Conversely, our data established a critical role of *Brd4* in the regulation of CD8^+^ T cells homeostasis and immunity. These T cells functions mainly rely on glucose uptake supported by Brd4. Thus, artificial manipulation of Brd4 may provide a novel therapeutic strategy for immunomodulation.

## Data Availability Statement

The original contributions presented in the study are publicly available. This data can be found here: https://www.ncbi.nlm.nih.gov/geo/query/acc.cgi?acc=GSE179492.

## Ethics Statement

The animal study was reviewed and approved by Institutional Animal Care and Use Committee of Sun Yet-Sen University. Written informed consent was obtained from the owners for the participation of their animals in this study.

## Author Contributions

ZP and HZ conceived and designed the study. ZP, JL, XM and YZ optimized the methods and analyzed high-throughput sequencing data. ZP, YZ, XM, MZ, ZS, YY, YC, YL, GW, FH, YQ, BX, WL, XZ, XH, TP, HX, and HZ performed the experiments. ZP and HZ proofread the whole manuscript. ZP, TP, HX, and HZ wrote, reviewed and edited the manuscript. All authors contributed to the article and approved the submitted version.

## Funding

This work was supported by the National Special Research Program of China for Important Infectious Diseases (2017ZX10202102 and 2018ZX10302103), the Important Key Program of Natural Science Foundation of China (81730060) and the Joint-innovation Program in Healthcare for Special Scientific Research Projects of Guangzhou (201803040002) to HZ and Guangzhou Science and Technology Project (201803010042)to XH. This work was also supported by National Natural Science Foundation of China (81971918), Shenzhen Science and Technology Program (Grant No. JSGG20200225150431472 and JCYJ20200109142601702), the Pearl River S&T Nova Program of Guangzhou (201806010118) and the Fundamental Research Funds for the Central Universities, Sun Yat-sen University (2021qntd43) to TP.

## Conflict of Interest

The authors declare that the research was conducted in the absence of any commercial or financial relationships that could be construed as a potential conflict of interest.

## Publisher’s Note

All claims expressed in this article are solely those of the authors and do not necessarily represent those of their affiliated organizations, or those of the publisher, the editors and the reviewers. Any product that may be evaluated in this article, or claim that may be made by its manufacturer, is not guaranteed or endorsed by the publisher.

## References

[1] Fischer A Deist F Hacein-Bey-Abina S André-Schmutz I de Saint Basile G de Villartay J-P. Severe Combined Immunodeficiency. A Model Disease for:Molecular Immunology and Therapy. Immunol Rev (2005) 203:98–109. 10.1111/j.0105-2896.2005.00223.x 15661024

[2] Surh CD Sprent J. Homeostasis of Naive and Memory T Cells. Immunity (2008) 29(6):848–62. 10.1016/j.immuni.2008.11.002 19100699

[3] Takada K Jameson SC. Naive T Cell Homeostasis: From Awareness of Space to a Sense of Place. Nat Rev Immunol (2009) 9(12):823–32. 10.1038/nri2657 19935802

[4] Sprent J Surh CD. Normal T Cell Homeostasis: The Conversion of Naive Cells Into Memory-Phenotype Cells. Nat Immunol (2011) 12(6):478–84. 10.1038/ni.2018 PMC3434123 21739670

[5] Masopust D Schenkel JM. The Integration of T Cell Migration, Differentiation and Function. Nat Rev Immunol (2013) 13(5):309–20. 10.1038/nri3442 23598650

[6] Carlson CM Endrizzi BT Wu J Ding X Weinreich MA Walsh ER. Kruppel-Like Factor 2 Regulates Thymocyte and T-Cell Migration. Nature (2006) 442(7100):299–302. 10.1038/nature04882 16855590

[7] Sebzda E Zou Z Lee JS Wang T Kahn ML. Transcription Factor KLF2 Regulates the Migration of Naive T Cells by Restricting Chemokine Receptor Expression Patterns. Nat Immunol (2008) 9(3):292–300. 10.1038/ni1565 18246069

[8] Kerdiles YM Beisner DR Tinoco R Dejean AS Castrillon DH DePinho RA. Foxo1 Links Homing and Survival of Naive T Cells by Regulating L-Selectin, CCR7 and Interleukin 7 Receptor. Nat Immunol (2009) 10(2):176–84. 10.1038/ni.1689 PMC2856471 19136962

[9] Desdin-Mico G Soto-Heredero G Mittelbrunn M. Mitochondrial Activity in T Cells. Mitochondrion (2018) 41:51–7. 10.1016/j.mito.2017.10.006 29032101

[10] Geltink RIK Kyle RL Pearce EL. Unraveling the Complex Interplay Between T Cell Metabolism and Function. Annu Rev Immunol (2018) 36:461–88. 10.1146/annurev-immunol-042617-053019 PMC6323527 29677474

[11] MacIver NJ Michalek RD Rathmell JC. Metabolic Regulation of T Lymphocytes. Annu Rev Immunol (2013) 31:259–83. 10.1146/annurev-immunol-032712-095956 PMC3606674 23298210

[12] Milasta S Dillon CP Sturm OE Verbist KC Brewer TL Quarato G. Apoptosis-Inducing-Factor-Dependent Mitochondrial Function Is Required for T Cell But Not B Cell Function. Immunity (2016) 44(1):88–102. 10.1016/j.immuni.2015.12.002 26795252 PMC4936487

[13] Mendoza A Fang V Chen C Serasinghe M Verma A Muller J. Lymphatic Endothelial S1P Promotes Mitochondrial Function and Survival in Naive T Cells. Nature (2017) 546(7656):158–61. 10.1038/nature22352 PMC5683179 28538737

[14] Ledderose C Liu K Kondo Y Slubowski CJ Dertnig T Denicolo S. Purinergic P2X4 Receptors and Mitochondrial ATP Production Regulate T Cell Migration. J Clin Invest (2018) 128(8):3583–94. 10.1172/JCI120972 PMC6063471 29894310

[15] Cham CM Gajewski TF. Glucose Availability Regulates IFN-Gamma Production and P70s6 Kinase Activation in CD8+ Effector T Cells. J Immunol (2005) 174(8):4670–7. 10.4049/jimmunol.174.8.4670 15814691

[16] Cham CM Driessens G O'Keefe JP Gajewski TF. Glucose Deprivation Inhibits Multiple Key Gene Expression Events and Effector Functions in CD8+ T Cells. Eur J Immunol (2008) 38(9):2438–50. 10.1002/eji.200838289 PMC3008428 18792400

[17] Vaeth M Maus M Klein-Hessling S Freinkman E Yang J Eckstein M. Store-Operated Ca2+ Entry Controls Clonal Expansion of T Cells Through Metabolic Reprogramming. Immunity (2017) 47(4):664–679.e6. 10.1016/j.immuni.2017.09.003 29030115 PMC5683398

[18] Wang R Dillon CP Shi LZ Milasta S Carter R Finkelstein D. The Transcription Factor Myc Controls Metabolic Reprogramming Upon T Lymphocyte Activation. Immunity (2011) 35(6):871–82. 10.1016/j.immuni.2011.09.021 PMC3248798 22195744

[19] Devaiah BN Gegonne A Singer DS. Bromodomain 4: A Cellular Swiss Army Knife. J Leukoc Biol (2016) 100(4):679–86. 10.1189/jlb.2RI0616-250R PMC5014741 27450555

[20] Shi J Vakoc CR. The Mechanisms Behind the Therapeutic Activity of BET Bromodomain Inhibition. Mol Cell (2014) 54(5):728–36. 10.1016/j.molcel.2014.05.016 PMC4236231 24905006

[21] Stanlie A Yousif AS Akiyama H Honjo T Begum NA. Chromatin Reader Brd4 Functions in Ig Class Switching as a Repair Complex Adaptor of Nonhomologous End-Joining. Mol Cell (2014) 55(1):97–110. 10.1016/j.molcel.2014.05.018 24954901

[22] Bao Y Wu X Chen J Hu X Zeng F Cheng J. Brd4 Modulates the Innate Immune Response Through Mnk2-Eif4e Pathway-Dependent Translational Control of IkappaBalpha. Proc Natl Acad Sci U.S.A. (2017) 114(20):E3993–4001. 10.1073/pnas.1700109114 PMC5441817 28461486

[23] Dey A Yang W Gegonne A Nishiyama A Pan R Yagi R. BRD4 Directs Hematopoietic Stem Cell Development and Modulates Macrophage Inflammatory Responses. EMBO J (2019) 38(7):e100293. 10.15252/embj.2018100293 PMC6443207 30842097

[24] Mele DA Salmeron A Ghosh S Huang HR Bryant BM Lora JM. BET Bromodomain Inhibition Suppresses TH17-Mediated Pathology. J Exp Med (2013) 210(11):2181–90. 10.1084/jem.20130376 PMC3804955 24101376

[25] Cheung K Lu G Sharma R Vincek A Zhang R Plotnikov AN. BET N-Terminal Bromodomain Inhibition Selectively Blocks Th17 Cell Differentiation and Ameliorates Colitis in Mice. Proc Natl Acad Sci USA (2017) 114(11):2952–7. 10.1073/pnas.1615601114 PMC5358349 28265070

[26] Cheung KL Zhang F Jaganathan A Sharma R Zhang Q Konuma T. Distinct Roles of Brd2 and Brd4 in Potentiating the Transcriptional Program for Th17 Cell Differentiation. Mol Cell (2017) 65(6):1068–80.e5. 10.1016/j.molcel.2016.12.022 28262505 PMC5357147

[27] Gegonne A Chen QR Dey A Etzensperger R Tai X Singer A. Immature CD8 Single-Positive Thymocytes Are a Molecularly Distinct Subpopulation, Selectively Dependent on BRD4 for Their Differentiation. Cell Rep (2018) 24(1):117–29. 10.1016/j.celrep.2018.06.007 PMC6298745 29972774

[28] Delmore JE Issa GC Lemieux ME Rahl PB Shi J Jacobs HM. BET Bromodomain Inhibition as a Therapeutic Strategy to Target C-Myc. Cell (2011) 146(6):904–17. 10.1016/j.cell.2011.08.017 PMC3187920 21889194

[29] Ott CJ Kopp N Bird L Paranal RM Qi J Bowman T. BET Bromodomain Inhibition Targets Both C-Myc and IL7R in High-Risk Acute Lymphoblastic Leukemia. Blood (2012) 120(14):2843–52. 10.1182/blood-2012-02-413021 PMC3466965 22904298

[30] Dang CV Le A Gao P. MYC-Induced Cancer Cell Energy Metabolism and Therapeutic Opportunities. Clin Cancer Res (2009) 15(21):6479–83. 10.1158/1078-0432.CCR-09-0889 PMC2783410 19861459

[31] Amorim S Stathis A Gleeson M Iyengar S Magarotto V Leleu X. Bromodomain Inhibitor OTX015 in Patients With Lymphoma or Multiple Myeloma: A Dose-Escalation, Open-Label, Pharmacokinetic, Phase 1 Study. Lancet Haematol (2016) 3(4):e196–204. 10.1016/S2352-3026(16)00021-1 27063978

[32] Berthon C Raffoux E Thomas X Vey N Gomez-Roca C Yee K. Bromodomain Inhibitor OTX015 in Patients With Acute Leukaemia: A Dose-Escalation, Phase 1 Study. Lancet Haematol (2016) 3(4):e186–95. 10.1016/S2352-3026(15)00247-1 27063977

[33] Zhu J Gaiha GD John SP Pertel T Chin CR Gao G. Reactivation of Latent HIV-1 by Inhibition of BRD4. Cell Rep (2012) 2(4):807–16. 10.1016/j.celrep.2012.09.008 PMC3523124 23041316

[34] Li Z Guo J Wu Y Zhou Q. The BET Bromodomain Inhibitor JQ1 Activates HIV Latency Through Antagonizing Brd4 Inhibition of Tat-Transactivation. Nucleic Acids Res (2013) 41(1):277–87. 10.1093/nar/gks976 PMC3592394 23087374

[35] Conrad RJ Fozouni P Thomas S Sy H Zhang Q Zhou MM. The Short Isoform of BRD4 Promotes HIV-1 Latency by Engaging Repressive SWI/SNF Chromatin-Remodeling Complexes. Mol Cell (2017) 67(6):1001–12 e6. 10.1016/j.molcel.2017.07.025 28844864 PMC5610089

[36] Li Z Mbonye U Feng Z Wang X Gao X Karn J. The KAT5-Acetyl-Histone4-Brd4 Axis Silences HIV-1 Transcription and Promotes Viral Latency. PloS Pathog (2018) 14(4):e1007012. 10.1371/journal.ppat.1007012 29684085 PMC5933813

[37] Kaya-Okur HS Janssens DH Henikoff JG Ahmad K Henikoff S. Efficient Low-Cost Chromatin Profiling With CUT&Tag. Nat Protoc (2020) 15(10):3264–83. 10.1038/s41596-020-0373-x PMC8318778 32913232

[38] Kochl R Thelen F Vanes L Brazao TF Fountain K Xie J. WNK1 Kinase Balances T Cell Adhesion Versus Migration In Vivo. Nat Immunol (2016) 17(9):1075–83. 10.1038/ni.3495 PMC4994873 27400149

[39] Barber DL Wherry EJ Ahmed R. Cutting Edge: Rapid *In Vivo* Killing by Memory CD8 T Cells. J Immunol (2003) 171(1):27–31. 10.4049/jimmunol.171.1.27 12816979

[40] Blattman JN Wherry EJ Ha SJ van der Most RG Ahmed R. Impact of Epitope Escape on PD-1 Expression and CD8 T-Cell Exhaustion During Chronic Infection. J Virol (2009) 83(9):4386–94. 10.1128/JVI.02524-08 PMC2668476 19211743

[41] Loven J Hoke HA Lin CY Lau A Orlando DA Vakoc CR. Selective Inhibition of Tumor Oncogenes by Disruption of Super-Enhancers. Cell (2013) 153(2):320–34. 10.1016/j.cell.2013.03.036 PMC3760967 23582323

[42] Walling BL Kim M. LFA-1 in T Cell Migration and Differentiation. Front Immunol (2018) 9:952. 10.3389/fimmu.2018.00952 29774029 PMC5943560

[43] Varga G Nippe N Balkow S Peters T Wild MK Seeliger S. LFA-1 Contributes to Signal I of T-Cell Activation and to the Production of T(h)1 Cytokines. J Invest Dermatol (2010) 130(4):1005–12. 10.1038/jid.2009.398 20072134

[44] Kondo N Ueda Y Kita T Ozawa M Tomiyama T Yasuda K. NDR1-Dependent Regulation of Kindlin-3 Controls High-Affinity LFA-1 Binding and Immune Synapse Organization. Mol Cell Biol (2017) 37(8). 10.1128/MCB.00424-16 PMC5376635 28137909

[45] Li D Molldrem JJ Ma Q. LFA-1 Regulates CD8+ T Cell Activation *via* T 2 Signal Pathways/2 Signal Pathways. J Biol Chem (2009) 284(31):21001–10. 10.1074/jbc.M109.002865 PMC2742865 19483086

[46] Milner JJ Toma C Quon S Omilusik K Scharping NE Dey A. Bromodomain Protein BRD4 Directs and Sustains CD8 T Cell Differentiation During Infection. J Exp Med (2021) 218(8):e20202512. 10.1084/jem.20202512 PMC8160575 34037670

[47] Ma EH Verway MJ Johnson RM Roy DG Steadman M Hayes S. Metabolic Profiling Using Stable Isotope Tracing Reveals Distinct Patterns of Glucose Utilization by Physiologically Activated CD8+ T Cells. Immunity (2019) 51(5):856–70.e5. 10.1016/j.immuni.2019.09.003 31747582

[48] Phan AT Doedens AL Palazon A Tyrakis PA Cheung KP Johnson RS. Constitutive Glycolytic Metabolism Supports CD8(+) T Cell Effector Memory Differentiation During Viral Infection. Immunity (2016) 45(5):1024–37. 10.1016/j.immuni.2016.10.017 PMC5130099 27836431

[49] Chang CH Qiu J O'Sullivan D Buck MD Noguchi T Curtis JD. Metabolic Competition in the Tumor Microenvironment Is a Driver of Cancer Progression. Cell (2015) 162(6):1229–41. 10.1016/j.cell.2015.08.016 PMC4864363 26321679

[50] Qiu J Villa M Sanin DE Buck MD O'Sullivan D Ching R. Acetate Promotes T Cell Effector Function During Glucose Restriction. Cell Rep (2019) 27(7):2063–74.e5. 10.1016/j.celrep.2019.04.022 31091446 PMC6544383

[51] Ho PC Bihuniak JD Macintyre AN Staron M Liu X Amezquita R. Phosphoenolpyruvate Is a Metabolic Checkpoint of Anti-Tumor T Cell Responses. Cell (2015) 162(6):1217–28. 10.1016/j.cell.2015.08.012 PMC4567953 26321681

[52] Khandekar D Tiriveedhi V. Role of BET Inhibitors in Triple Negative Breast Cancers. Cancers (Basel) (2020) 12(4):784–99. 10.3390/cancers12040784 PMC7226117 32218352

[53] Liu C Ma X Liu B Chen C Zhang H. HIV-1 Functional Cure: Will the Dream Come True? BMC Med (2015) 13:284. 10.1186/s12916-015-0517-y 26588898 PMC4654816

[54] Guangming Li ZZ Reszka-Blanco N Li F Chi L Jianping M Jerry J. Specific Activation *In Vivo* of HIV-1 by a Bromodomain Inhibitor From Monocytic Cells in Humanized Mice Under Antiretroviral Therapy. J Viol (2019) 93:e00233–19. 10.1128/JVI.00233-19 PMC6613761 30971469

[55] Ma X Yang T Luo Y Wu L Jiang Y Song Z. TRIM28 Promotes HIV-1 Latency by SUMOylating CDK9 and Inhibiting P-TEFb. Elife (2019) 8:e42426. 10.7554/eLife.42426 PMC6361614 30652970

[56] Zhangping He SJ Yang T Chen J Huang F Zhang W Peng Z. PIWIL4 Maintains HIV-1 Latency by Enforcing Epigenetically Suppressive Modifications on the 5′ Long Terminal Repeat. J Viol (2020) 94:e01923–19. 10.1128/JVI.01923-19 PMC7199406 32161174

[57] Ma X Chen T Peng Z Wang Z Liu J Yang T. Histone Chaperone CAF-1 Promotes HIV-1 Latency by Leading the Formation of Phase-Separated Suppressive Nuclear Bodies. EMBO J (2021) 40(10):e106632. 10.15252/embj.2020106632 33739466 PMC8126954

[58] Georgiev P Wang Y Muise ES Bandi ML Blumenschein W Sathe M. BET Bromodomain Inhibition Suppresses Human T Cell Function. Immunohorizons (2019) 3(7):294–305. 10.4049/immunohorizons.1900037 31356159

[59] Gibbons HR Mi DJ Farley VM Esmond T Kaood MB Aune TM. Bromodomain Inhibitor JQ1 Reversibly Blocks IFN-Gamma Production. Sci Rep (2019) 9(1):10280. 10.1038/s41598-019-46516-x 31311960 PMC6635431

[60] Wein AN McMaster SR Takamura S Dunbar PR Cartwright EK Hayward S. CXCR6 Regulates Localization of Tissue-Resident Memory CD8 T Cells to the Airways. J Exp Med (2019) 216(12):2748–62. 10.1084/jem.20181308 PMC6888981 31558615

[61] McNamara HA Y C Wagle MV Sontani Y Roots CM Miosge LA. Up-Regulation of LFA-1 Allows Liver-Resident Memory:T Cells to Patrol and Remain in the Hepatic Sinusoids. Sci Immunol (2017) 2:eaaj1996. 10.1126/sciimmunol.aaj1996 28707003 PMC5505664

[62] Franciszkiewicz K Le Floc'h A Boutet M Vergnon I Schmitt A Mami-Chouaib F. CD103 or LFA-1 Engagement at the Immune Synapse Between Cytotoxic T Cells and Tumor Cells Promotes Maturation and Regulates T-Cell Effector Functions. Cancer Res (2013) 73(2):617–28. 10.1158/0008-5472.CAN-12-2569 23188505

[63] Amsen D van Gisbergen Kpjm Hombrink P van Lier RAW. Tissue-Resident Memory T Cells at the Center of Immunity to Solid Tumors. Nat Immunol (2018) 19(6):538–46. 10.1038/s41590-018-0114-2 29777219

[64] Borck PC Guo LW Plutzky J. BET Epigenetic Reader Proteins in Cardiovascular Transcriptional Programs. Circ Res (2020) 126(9):1190–208. 10.1161/CIRCRESAHA.120.315929 PMC8111334 32324495

[65] Kannan-Sundhari A Abad C Maloof ME Ayad NG Young J Liu XZ. Bromodomain Protein BRD4 Is Essential for Hair Cell Function and Survival. Front Cell Dev Biol (2020) 8:576654. 10.3389/fcell.2020.576654 33015071 PMC7509448

[66] Korb E Herre M Zucker-Scharff I Darnell RB Allis CD. BET Protein Brd4 Activates Transcription in Neurons and BET Inhibitor Jq1 Blocks Memory in Mice. Nat Neurosci (2015) 18(10):1464–73. 10.1038/nn.4095 PMC4752120 26301327

