# Supplementary material for: Brd4 Regulates the Homeostasis of CD8+ T-Lymphocytes and Their Proliferation in Response to Antigen Stimulation
Source: Front Immunol. 2021 Aug 26;12:728082. doi: 10.3389/fimmu.2021.728082 (PMC8427756; doi:10.3389/fimmu.2021.728082)
Supplement: Supplementary file 1 [file DataSheet_1.docx]

**Supplementary Figures**


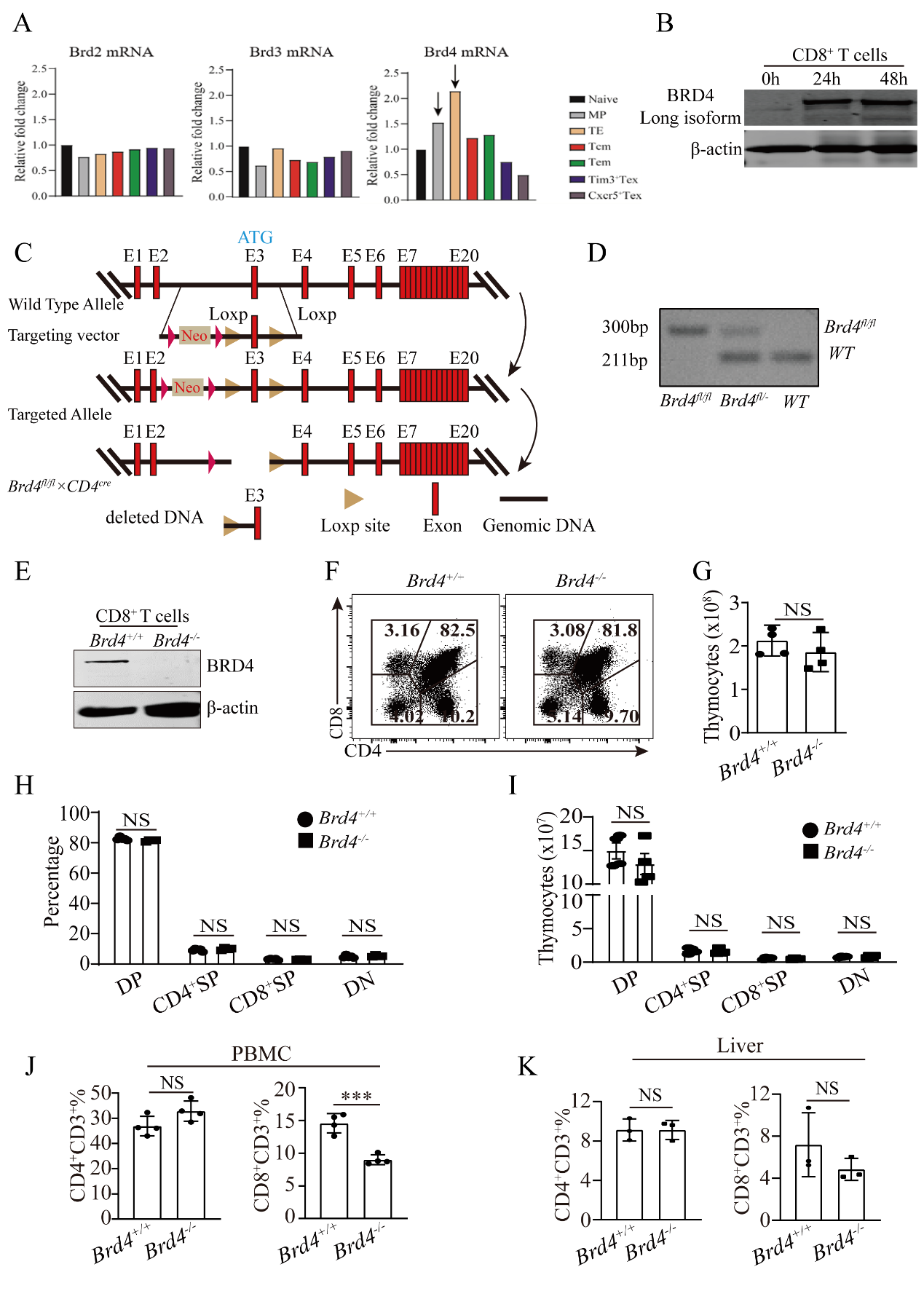


**Supplemental Figure 1:** **Thymocytes normally develop in *Brd4*^-/-^ mice.** (A) Gene expression of BET family proteins in distinct CD8^+^ T-cell subsets from public database and the expression value in naïve CD8^+^ T cell set as 1. (B) Immunoblotting analysis of BRD4 in CD8^+^ T cells at distinct time point after activation. (C) Schematic representation of *Brd4^fl/fl^* mice construction. The exon organization of the *Brd4* locus (top). Targeting vector contained exon 3 flanked by LoxP sites (yellow) and a neomycin resistance gene flanked by FRT. *CD4^Cre^*-mediated recombination results in the deletion of exon 3, which leads to loss of BRD4 expression (bottom). (D) Genotyping confirmation of *Brd4^fl/fl^* mice. (E) Immunoblotting analysis of Brd4 protein in CD8^+^ T cells from *Brd4*^+/+^ and *Brd4^-/-^* mice. (F) Flow cytometry showing the expression of CD4 and CD8 in thymocytes. (G) The percentage of thymocyte subsets. (H) The quantification of total thymocytes. (I) The quantification of absolute thymocytes subsets. (J) The percentage of CD4^+^ T (left) and CD8^+^ T cells (right) in PBMC. (K) The percentage of CD4^+^ T (left) and CD8^+^ T cells (right) in liver. Two-tailed unpaired t-test was used to analyze two independent groups. Results were indicated as mean ± sem (error bars). *p ＜0.05; **p ＜0.01; ***p ＜ 0.001. NS, not significant. Each group includes at least 3 mice and each experiment repeats more than 2 times.


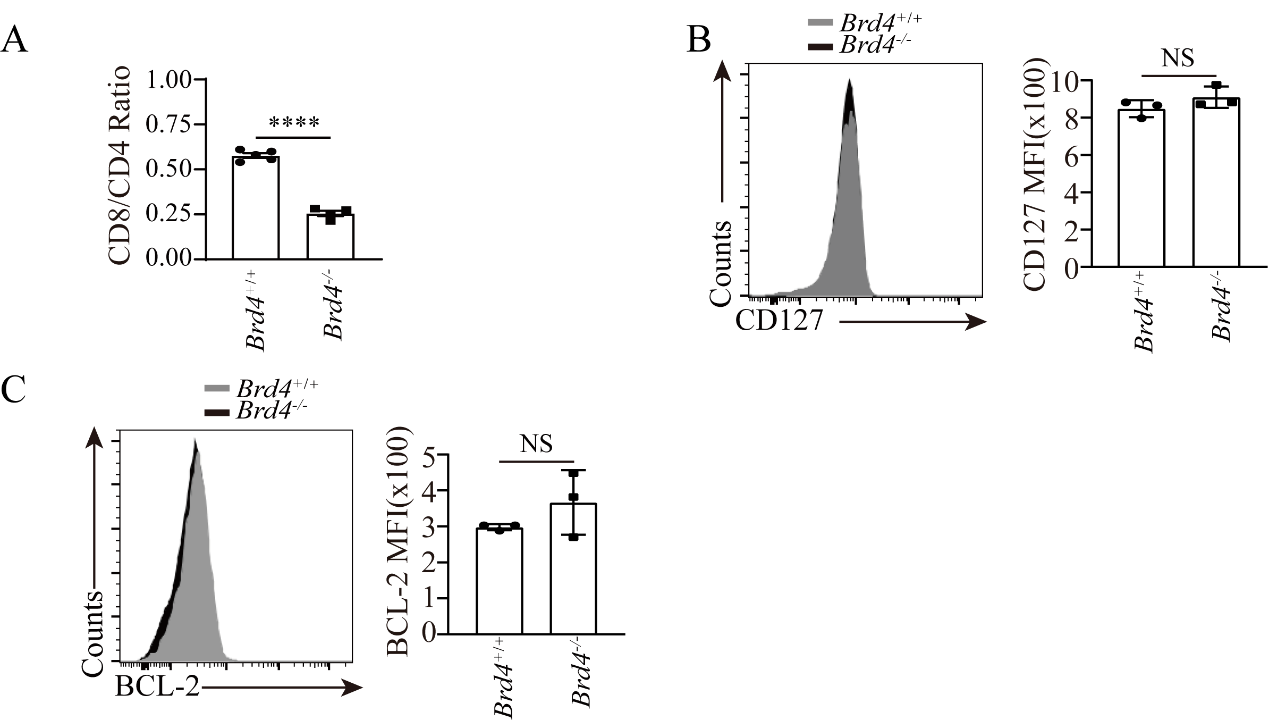


**Supplemental Figure 2: Analysis of IL-7Rα and BCL-2 expression.** (A) The ratio of CD8 to CD4 in spleen. (B) Flow cytometry showing CD127 expression in *Brd4^+/+^* and *Brd4*^-/-^ naïve CD8^+^ T cells (left) and the quantification of CD127 MFI (right). (C) Flow cytometry analysis of BCL-2 expression in *Brd4^+/+^* and *Brd4*^-/-^ naïve CD8^+^T cells(left) and the quantification of BCL-2 MFI (right). Two-tailed unpaired t-test was used to analyze two independent groups. Results were indicated as mean ± sem (error bars). ****p ＜ 0.0001. NS, not significant. Each group includes at least 3 mice and each experiment repeats more than 2 times.


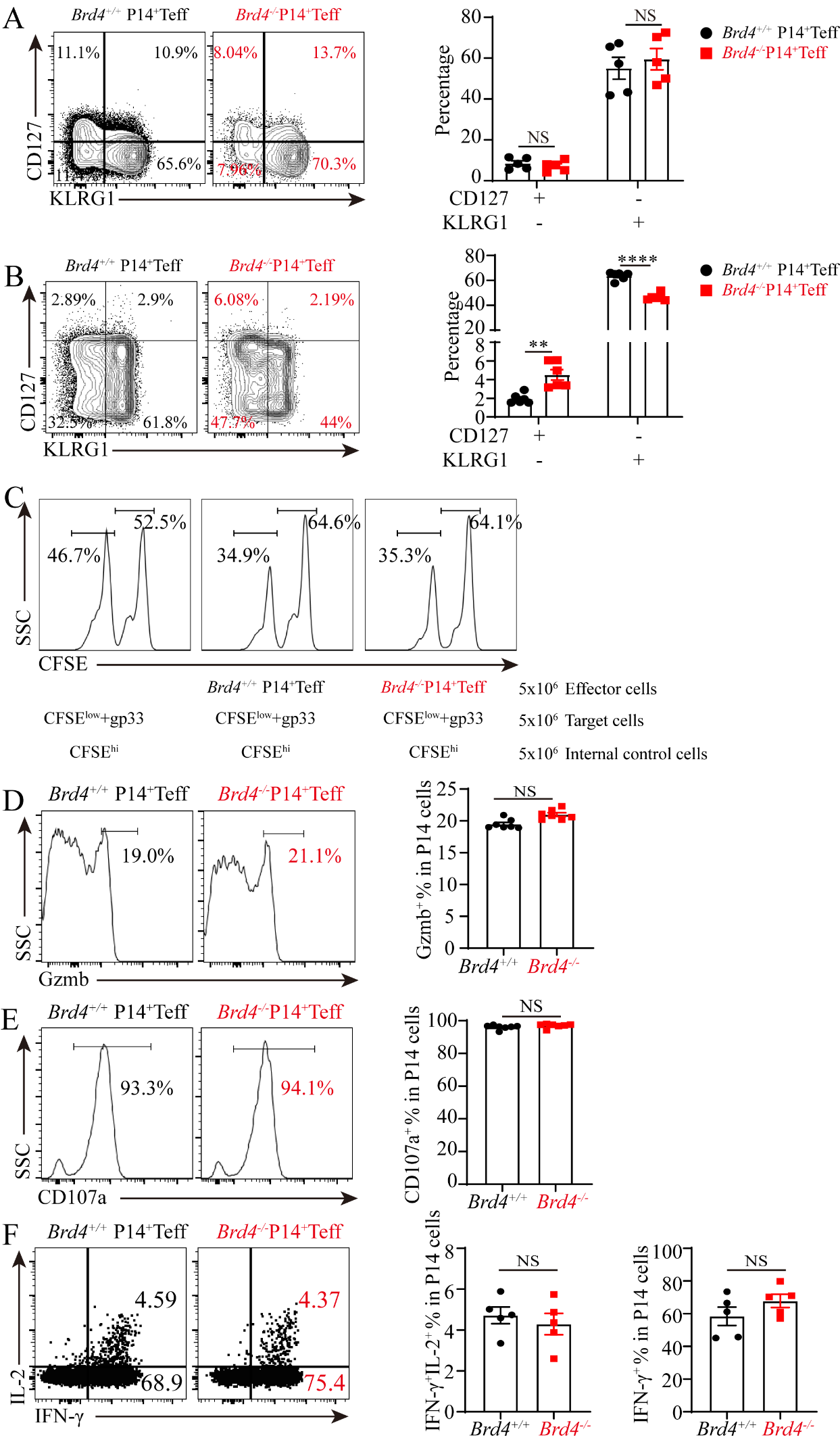


**Supplemental Figure 3: The phenotype and function characteristic of *Brd4^+/+^* and *Brd4^-/-^* effector P14^+^CD8^+^ T cells.** (A) 5000 *Brd4^+/+^*(CD45.1^+^CD45.2^+^) and *Brd4*^-/-^(CD45.1^-^CD45.2^+^) naive P14^+^CD8^+^ T cells were mixed and transferred into recipient (CD45.1^+^CD45.2^-^) to explore the effect of Brd4 deletion on CD8^+^ T cells differentiation and function. The surface expression of CD127 and KLRG1 on donor *Brd4*^+/+^ and *Brd4*^-/-^ P14^+^CD8^+^ T cell from LCMV-arm infected host at day 8 (left) and the quantification of the frequency of memory precursors subset (CD127^+^ KLRG1^-^) and terminal differentiation subset (CD127^-^KLRG1^+^) within donor effector P14 cells population (right). (B) *1000 Brd4^+/+^*(CD45.1^+^CD45.2^+^) and *Brd4^-/-^*(CD45.1^-^CD45.2^+^) naive P14^+^CD8^+^ T cells were mixed and transferred into recipient (CD45.1^+^CD45.2^-^) to explore the effect of *Brd4* deletion on CD8^+^ T cells differentiation and function. The surface expression of CD127 and KLRG1 on donor *Brd4^+/+^* and *Brd4^-/-^* P14^+^CD8^+^ T cell from LCMV-arm infected host at day 8 (left) and the quantification of the frequency of memory precursors subset (CD127^+^ KLRG1^-^) and terminal differentiation subset (CD127^-^KLRG1^+^) within donor effector P14 cells population (right). (C) Representative result of in vivo *Brd4^+/+^* and *Brd4^-/-^* P14^+^CD8^+^ T cells killing assay. CFSE^lo^ (specific) and CFSE^hi^ (non-specific) target cells were mixed at a 1:1 ratio and the co-transferred with effector cells into naïve mice. Histograms are gated on CD45.2^+^ target cells in the spleen. Numbers represent the percentage of target cells killed. (D) Flow cytometry showing the production of granzyme B in donor *Brd4^+/+^* and *Brd4^-/-^* P14^+^CD8^+^ T cells from infection host at day 8 (left) and the frequency of Gzmb^+^ P14^+^CD8^+^ T cells (right). (E) Flow cytometry showing the representative degranulation result based on CD107a expression in donor *Brd4^+/+^* and *Brd4^-/-^* P14^+^CD8^+^ T cells from infection host at day 8 (left) and the frequency of CD107a^+^ P14^+^CD8^+^ T cells (right).

(F) Flow cytometry showing IFN-γ and IL-2 production in donor *Brd4^+/+^* and *Brd4^-/-^* P14^+^CD8^+^ T cells (left) and the proportion of IFN-γ^+^ and IFN-γ^+^ IL-2^+^ donor P14^+^CD8^+^ T cells (right). Paired Student’s *t*-test was used for data analysis. Results were indicated as mean ± sem (error bars). **p ＜0.01; ***p ＜ 0.001. NS, not significant. Each group includes at least 6 mice.


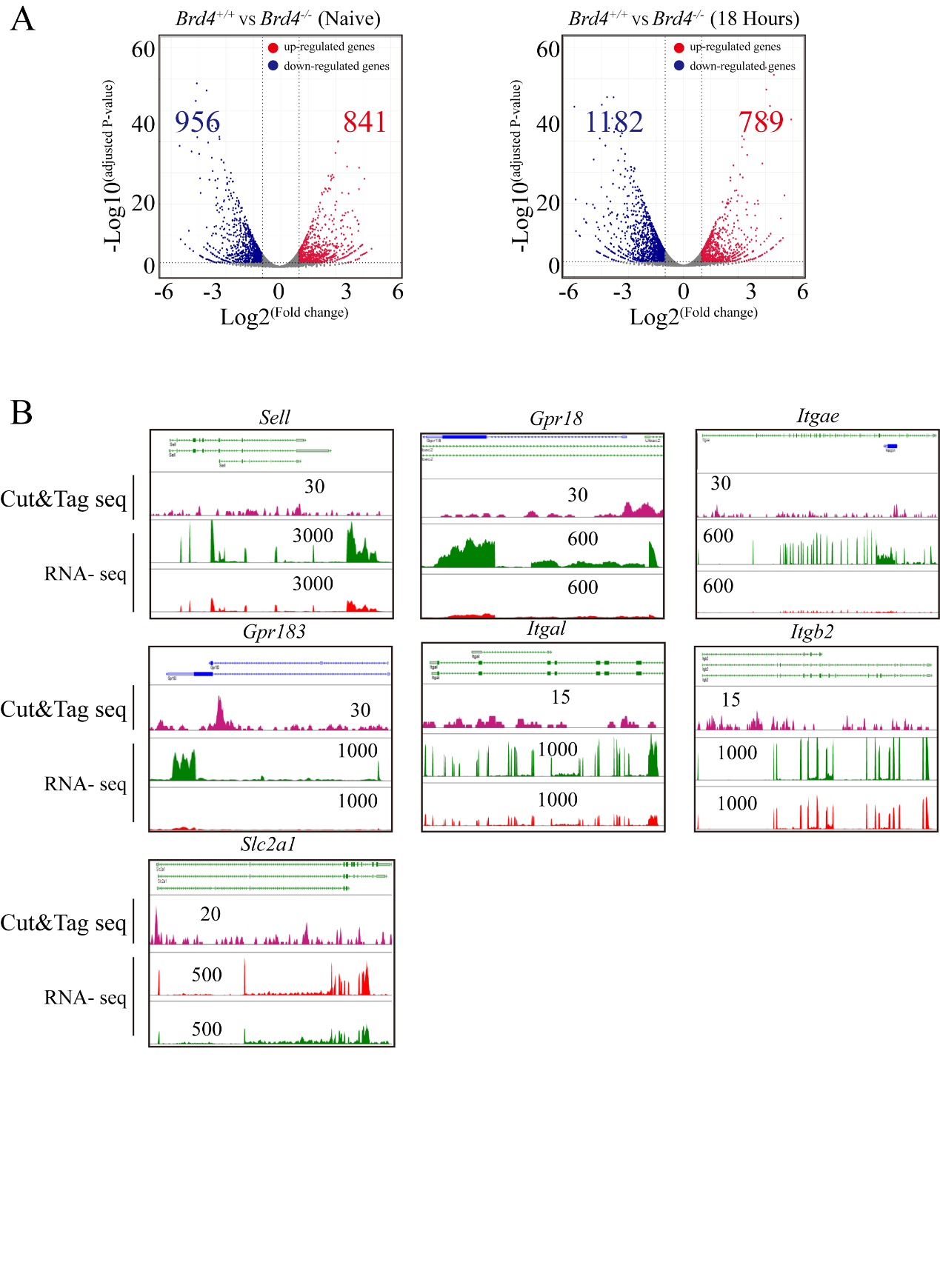


**Supplemental Figure 4: Profiling the binding sites of BRD4 in indicated gene locus.** (A) *Brd4*^+/+^ and *Brd4^-/-^* naïve CD8^+^ T cells or in vitro-activated CD8^+^ T cells were used to generate RNA-seq library. Volcano plot showing the differentially expressed genes in naïve and activated CD8^+^ T cells. (B) Profiling the binding sites of BRD4 in indicated gene locus.


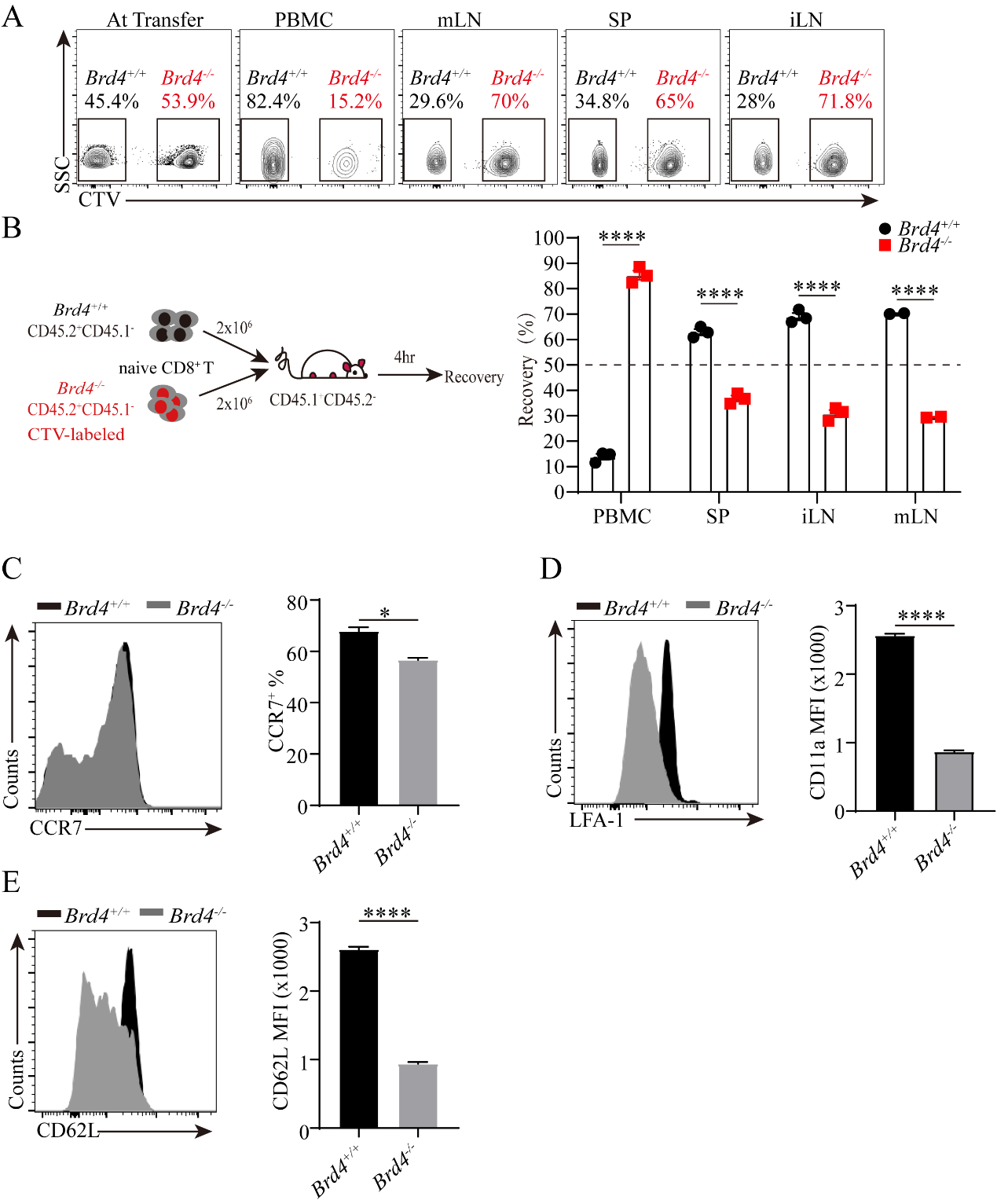
 **Supplemental Figure 5: Homing receptor expression in peripheral blood-derived CD8^+^ T cells.** (A) Flow cytometry showing the representative results of in vivo homing of *Brd4*^+/+^ and *Brd4*^-/-^ naïve CD8^+^ T cells at 4 h post-transfer. (B) The relative proportion of donor naïve CD8^+^ T cells in indicated tissue at 4 h. (C) Flow cytometry showing CCR7 expression in naïve CD8^+^ T cells from *Brd4*^+/+^ and *Brd4*^-/-^ mice. (D) Flow cytometry showing LFA-1 expression in naïve CD8^+^ T cells from *Brd4*^+/+^ and *Brd4*^-/-^ mice. (E) Flow cytometry showing CD62L expression in naïve CD8^+^ T cells from *Brd4*^+/+^ and *Brd4*^-/-^ mice. Two-tailed unpaired t-test was used to analyze two independent groups, while paired Student’s t-test was used when sample being compared from same mouse. Results were indicated as mean ± sem (error bars). *p ＜0.05; ***p ＜ 0.001; ***p ＜ 0.001. Each group includes at least 3 mice.


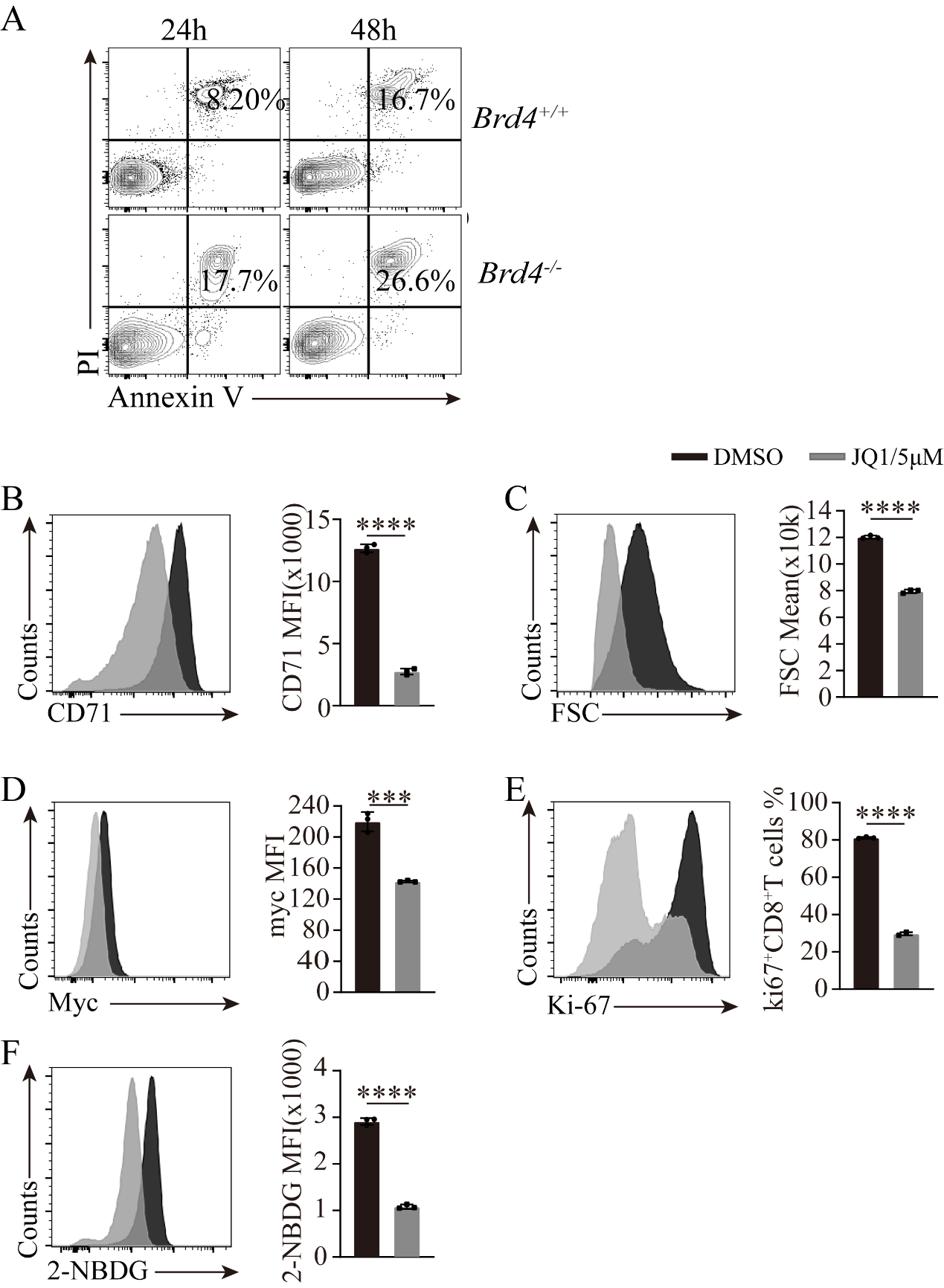


**Supplemental Figure 6: JQ1 treatment impairs CD8^+^ T cells activation.** (A) Flow cytometry showing the apoptosis staining of *Brd4^-/-^* and *Brd4^+/+^* CD8^+^ T cells upon activation. (B) Naïve CD8^+^ T cells were activated with anti-CD3 and anti-CD28 antibody in vitro for 24 h in the presence or absence of JQ1. Flow cytometry analysis showing the glucose uptake in CD8^+^ T cells after activation for 24 h. (C) Cell size analysis by flow cytometry after activation for 24 h. (D) Flow cytometry analysis showing the expression of CD71 after activation for 24 h. (E) Intracellular staining for Myc detection after JQ1 or DMSO treatment for 24 h. (F) Intracellular staining for ki-67 expression in CD8^+^ T cells after activation for 24 h. Two-tailed unpaired t-test was used to analyze two independent groups. Results were indicated as mean ± sem (error bars). ***p ＜0.001; *****p ＜ 0.0001.


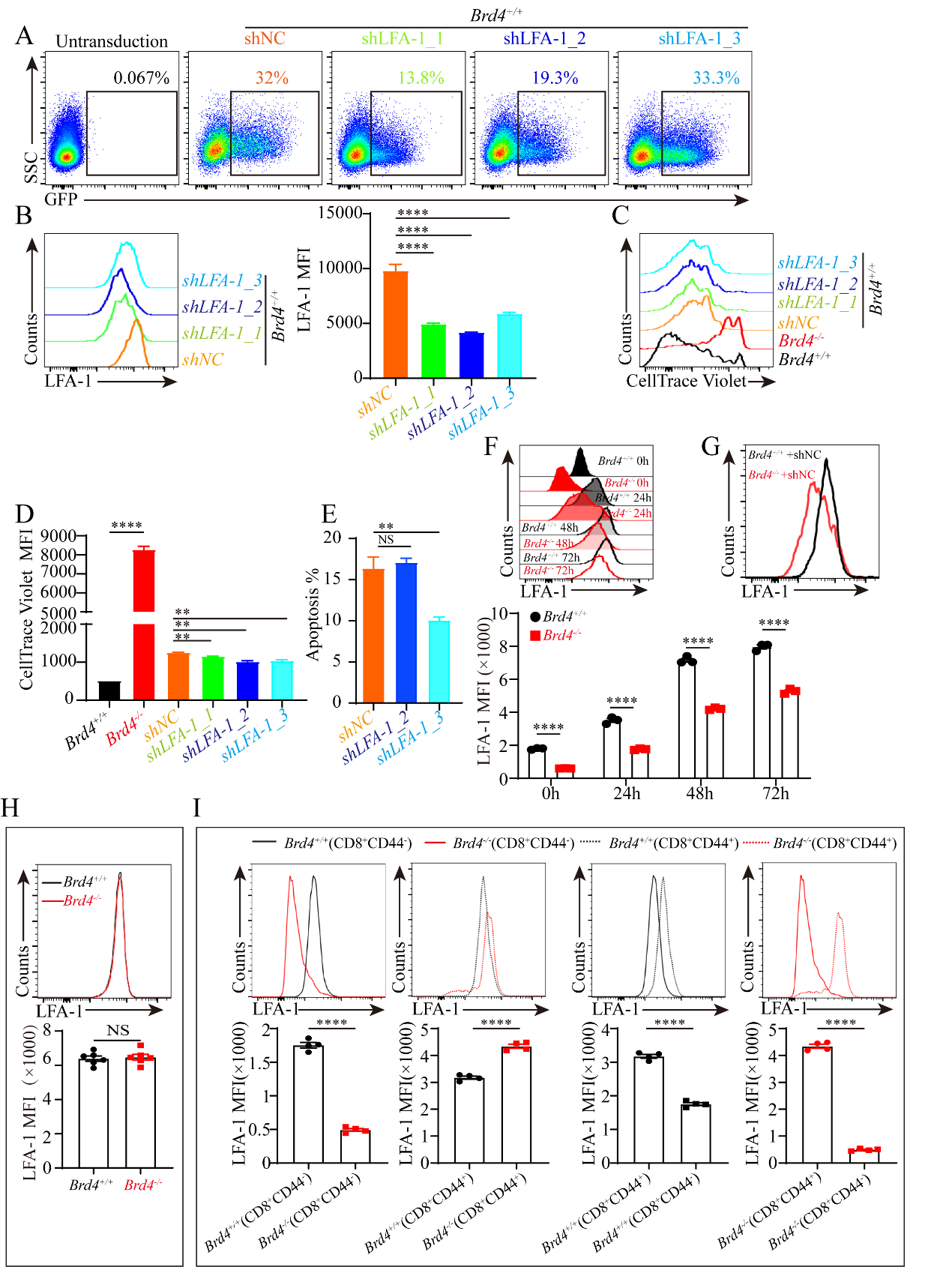
**Supplemental Figure 7: The evaluation of the effect of LFA-1 knockdown on CD8^+^ T cells proliferation and survival.** (A) Flow cytometry showing the representative results of transduction efficiency of shRNA-expressing retrovirus. (B) Flow cytometry showing the expression of LFA-1 in CD8^+^ T cells transduced with distinct shRNA (left) and the quantification of LFA-1 MFI in in CD8^+^ T cells transduced with distinct shRNA (right). (C) Flow cytometry showing the proliferation of CD8^+^ T cells transduced with different shRNA-expressing vectors. (D) The quantification of CellTrace Violet MFI of CD8^+^ T cells as in (C). (E) The proportion of apoptotic cells within CD8^+^ T cells transduced with indicated shRNA-expressing vector. (F) Flow cytometry showing the dynamic expression of LFA-1 in naïve *Brd4*^+/+^ and *Brd4*^-/-^ CD8^+^ T cells after TCR stimulation at distinct time point (top) and the quantification of LFA-1 MFI in distinct time point (bottom). (G) Flow cytometry showing the expression of LFA-1 in shNC-transduced *Brd4*^+/+^ and *Brd4*^-/-^ CD8^+^ T cells. (H) Flow cytometry showing the expression of LFA-1 in donor *Brd4^+/+^* and *Brd4^-/-^* P14^+^CD8^+^ T cells recovered from infection recipient mice at day 8 (left) and the quantification of LFA-1 in donor *Brd4^+/+^* and *Brd4^-/-^* P14^+^CD8^+^ T cells (right). (I) Flow cytometry showing the expression of LFA-1 in indicated CD8^+^ T cells (top) and the quantification of LFA-1 MFI in indicated groups (bottom). Two-tailed unpaired t-test was used to analyze two independent groups. To compare multiple groups, we used an analysis of variance (ANOVA). Results were indicated as mean ± sem (error bars). **p ＜0.01; ***p ＜ 0.001. NS, not significant. Each experiment has three biologic replicates at least.
